# “Assessing Today for a Better Tomorrow”: An observational cohort study about quality of care, mortality and morbidity among newborn infants admitted to neonatal intensive care in Guinea

**DOI:** 10.1371/journal.pone.0254938

**Published:** 2021-08-30

**Authors:** Maria Bea Merscher Alves, N’Fanly Conté, Boubacar Diallo, Moustapha Mamadou, Albert Delamou, Oliver John, Stefanie von Felten, Ibrahima Sory Diallo, Matthias Roth-Kleiner

**Affiliations:** 1 Pediatric and Neonatal Intensive Care Unit, Children’s Research Center, University Children’s Hospital Zurich, University of Zurich, Zurich, Switzerland; 2 Clinic of Neonatology, Institute of Nutrition and Child Health, Conakry, Guinea; 3 Master Program in Biostatistics, University of Zurich, Zurich, Switzerland; 4 Department of Biostatistics, Epidemiology, Biostatistics and Prevention Institute, University of Zurich, Zurich, Switzerland; 5 Department Woman-Mother-Child, Clinic of Neonatology, Lausanne University Hospital and University of Lausanne, Lausanne, Switzerland; Federal University of Sergipe, BRAZIL

## Abstract

**Background:**

Neonatal mortality in Guinea accounts for about 30% of all fatalities in children younger than five years. Countrywide, specialized neonatal intensive care is provided in one single clinic with markedly limited resources. To implement targeted measures, prospective data on patient characteristics and factors of neonatal death are needed.

**Objective:**

To determine the rates of morbidity and mortality, to describe clinical characteristics of admitted newborns requiring intensive care, to assess the quality of disease management, and to identify factors contributing to neonatal mortality.

**Methods:**

Prospective observational cohort study of newborns admitted to the hospital between mid-February and mid-March 2019 after birth in other institutions. Data were collected on maternal/prenatal history, delivery, and in-hospital care via convenience sampling. Associations of patient characteristics with in-hospital death were assessed using cause-specific Cox proportional-hazards models.

**Results:**

Half of the 168 admitted newborns underwent postnatal cardiopulmonary resuscitation. Reasons for admission included respiratory distress (49.4%), poor postnatal adaptation (45.8%), prematurity (46.2%), and infections (37.1%). 101 newborns (61.2%) arrived in serious/critical general condition; 90 children (53.9%) showed clinical signs of neurological damage. Quality of care was poor: Only 59.4% of the 64 newborns admitted with hypothermia were externally heated; likewise, 57.1% of 45 jaundiced infants did not receive phototherapy. Death occurred in 56 children (33.3%) due to birth asphyxia (42.9%), prematurity (33.9%), and sepsis (12.5%). Newborns in serious/critical general condition at admission had about a fivefold higher hazard to die than those admitted in good condition (HR 5.21 95%-CI 2.42–11.25, p = <0.0001). Hypothermia at admission was also associated with a higher hazard of death (HR 2.00, 95%-CI 1.10–3.65, p = 0.023).

**Conclusion:**

Neonatal mortality was strikingly high. Birth asphyxia, prematurity, and infection accounted for 89.3% of death, aggravated by poor quality of in-hospital care. Children with serious general condition at admission had poor chances of survival. The whole concept of perinatal care in Guinea requires reconsideration.

## Introduction

The neonatal period, defined as the first 28 days of life, is the most vulnerable time for an infant’s survival. Worldwide, approximately three million newborn deaths are recorded each year, the majority, though, die unregistered at home [1]. Eight Millennium Development Goals (MDG) were defined by the United Nations (UN) in 2000, with the fourth goal focusing on the reduction of the under-five-mortality rate by two-thirds between 1990 and 2015 [2]. In 2016, the Sustainable Development Goals (SDG) were developed by the UN as successors to the MDG with the third SDG focusing on improving child and maternal health. Specifically, target 3.2.2 of the SDG calls for a reduction in neonatal mortality in all countries to twelve or fewer deaths per 1,000 live births by 2030 [3]. Due to national and international achievements, the global neonatal mortality rate (NMR) fell by 51 percent from 37 deaths per 1,000 live births in 1990 to 18 in 2017; however, neonatal deaths still account for 47 percent of worldwide under-five deaths [4]. Globally, half of the infants dying within their first 28 days lose their lives within the first day of life, two-thirds within the first week [5]. Despite a declining global NMR, marked disparities in the level of neonatal deaths exist across regions, with a low NMR of fewer than twelve deaths per 1,000 live births in developed countries and a persistently high NMR of 28 deaths per 1,000 live births in sub-Saharan Africa [6].

The Republic of Guinea is a poverty-stricken West-African country, with a high burden of disease, an ill-equipped public healthcare system, underpaid health workers, and poor sanitation [7]. Guinea decreased its NMR by half from 62 deaths per 1,000 live births in 1990 to 31 deaths per 1,000 live births in 2018; still accounting for 14,000 newborn deaths annually and representing almost one-third of all fatalities in children younger than five years [6]. The discrepancy between the current NMR and the proclaimed SDG target highlights the urgency of diminishing newborn death in Guinea. Preventing newborn deaths specifically is ever more important not only because of the increasing proportion of neonatal death among under-five fatalities but also because the required health interventions that are needed to address the main causes of neonatal mortality mostly differ from those required to address other under-five deaths [8].

In 2016, the major causes of neonatal death worldwide were birth asphyxia (BA, 31%), prematurity (27%), and sepsis (21%) [9, 10]. Importantly, most neonatal deaths are preventable by simple and affordable interventions, such as clean birth conditions, birth attendants trained in basic newborn resuscitation, postpartum bag and mask respiratory support, access to antibiotics, and external heat sources, such as maternal skin-to-skin contact [11]. Antenatal care and skilled support during birth are infrequent in Guinea and depend markedly on the household wealth as well as the residential proximity to urban areas [12]. Less than half of the effectively required nurses and midwives for maternal and neonatal health services are currently available in Guinea, and the existing health workforce is concentrated in large cities due to urbanization [13]. Fully functioning emergency obstetric and neonatal care units are only available in three percent of all health facilities, all of those being reference hospitals [14]. Nationwide, specialized neonatal intensive care at university-level is only provided in a single clinic in the capital, operating under the restraint of pronounced resource scarcity. Since 2015, the maternity ward of the joined university hospital has been closed for construction. Therefore, all neonatal admissions are outborn babies referred postnatally to the neonatal unit without assistance during transfer and often with considerable delay, increasing their risk of death [15].

To facilitate the implementation of targeted specific measures and increase their respective impact, it is crucial to identify potential causal factors leading to neonatal morbidity and mortality. The objectives of this study were to: (1) determine the rates of neonatal morbidity and mortality at the only specialized neonatal intensive care unit (NICU) in Guinea, (2) describe the clinical characteristics of the hospitalized patient population, (3) document and assess the currently available care, as well as (4) identify potential factors contributing to neonatal death. The overall aim of this study was to gain a better understanding of the status quo of newborn healthcare in Guinea in order to identify areas of improvement and contribute to the decrease in the NMR as envisioned by the SDG.

## Materials and methods

A prospective observational cohort study was conducted at the Institute of Nutrition and Child Health (Institut de Nutrition et de Santé de l’Enfant, INSE) of all admitted newborns and their follow-up until discharge or death during a 30-day period from February 15^th^ to March 16^th^ 2019. Ethical approval was granted from the Comité National d’Ethique pour la Recherche en Santé (CNERS) in Guinea (Ref. 035/CNERS/19). The written informed consent was signed by a parent or legal guardian prior to collecting and analyzing data.

### Hospital setting

INSE is adjacent to the large public university hospital Donka and functions as the only specialized NICU in Guinea with a theoretical catchment area of the whole country with a population of 13.4 million inhabitants [16]. However, very few newborns outside the wider Conakry area, with a population of about 1.9 Mio inhabitants, are presented at INSE [17]. The unique shape of the capital city, a slim peninsula with all important facilities located at the coastal end and only two main roads connecting all suburbs with downtown Conakry, causes major traffic congestion and access difficulties to the medical care center. The Donka University Hospital has not offered obstetric services since 2015 due to renovation of the maternity ward. Therefore, all admitted neonates were born in other facilities or at home, and, consequently, had to be transported postnatally to INSE.

On average, 4500 children are hospitalized annually at INSE, more than half of them in the neonatal department. Reasons for neonatal admissions include prematurity and low birth weight (LBW, <2500 g), poor postnatal adaptation, out-of-hospital reanimation, respiratory distress, bleeding, fever or other signs of infection, poor feeding, and congenital malformations. The architectural structure of the neonatal department offers only very limited space with overcrowded rooms and patients often sharing beds. The critical care unit accommodates 15–20 newborns. Further 25–30 newborn infants, once stabilized, are cared for in two more rooms. In each unit, one pediatrician is in charge supervising a general practitioner and an intern doctor from 9 am to 3 pm. After 3 pm, one general practitioner and two medical students assume the night shift for the whole neonatal department. Two specialized neonatologists work at the facility as consultants during daytime. The Kangaroo Mother Care (KMC) unit houses four premature newborns and their mothers, under the surveillance of a nurse teaching skin-to-skin-nursing. The other mothers stay in self-supply dormitories on the premises. The hospital is ill-equipped and underfunded. There had been no air conditioning at the time of the study and power outages were frequent. Solar panels power emergency lighting, oxygen extractors, and the one available phototherapy lamp. Equipment is of inferior quality and deteriorates quickly due to heat, dust, humidity, and power instability. Infants sleep in plastic cradles or makeshift wooden boxes. All medication and medical supplies have to be purchased by the parents after medical prescription, even in cases of emergency. Breastfeeding is encouraged according to the newborns’ general condition and gestational age; otherwise, the newborns are fed with expressed breastmilk or formula milk via nasogastric tube, cup, or spoon. Premature or hypothermic newborns are placed in plastic bags and externally heated under a shared radiant warmer, or with the help of warm water bottles.

### Management of admitted newborns

Managing admitted newborns, medical staff followed the guidelines of Advanced Neonatal Care by Médecins Sans Frontières (MSF) who had trained local personnel over several occasions until 2016. These guidelines are based on the World Health Organization (WHO) Essential Newborn Care guideline [18]. Vital parameters (axillary temperature, heart rate, respiratory rate, and peripheral oxygen saturation) were obtained using a digital thermometer and pulse oximeter, respectively. The body weight and findings of a full-body exam were recorded at admission. Respiratory distress was categorized as mild, moderate, or severe using the Silverman Score [19]. Interventions included airway suction for clearance, positive pressure ventilation for non-breathing infants with an Ambu^®^ breathing bag, and oxygen therapy using oxygen concentrators with their output shared via Y-pieces of nasal cannula. Continuous positive airway pressure (CPAP), intubation, and mechanical ventilators were not available to assist newborns with respiratory distress. Nota bene, all pharmacological treatments were only administered if they were available, and had been purchased by the parents in the in-hospital or external pharmacies. Patients were routinely administered 1 mg of intramuscular Vitamin K if not yet injected in the obstetric clinic. Caffeine citrate (loading dose 10 mg/kg/day, maintenance dose 5 mg/kg/day) was given to premature newborns with gestational age <34 weeks or once apnea was noted. Cardiac arrest and bradycardia defined as heart rate below 60 beats/minute were treated with bag and mask ventilation and manual chest compressions. Anemia was defined as severe when the hemoglobin level was below 14 mg/dl. In case of severe anemia or active bleeding, blood transfusions with 20–25 ml/kg of concentrate red cells over 180–240 minutes were given. Neonatal jaundice was evaluated according to the Modified Kramer’s Scale as well as serum bilirubin levels, if available [20]. Phototherapy was used to treat jaundice with four hour-long sessions at a time; however, light therapy was often withheld due to hyperthermia. Intravenous antibiotic treatment was administered to newborns, for at least 48 hours, if either sepsis was being suspected (based on clinical signs and/or laboratory results), or if infection could not be excluded (unavailable prenatal history or laboratory results). No hemocultures were available. Ampicillin 50 mg/kg/day and gentamycin 5 mg/kg/day were administered to all patients without clinical signs of sepsis but suspected infection; ampicillin 100 mg/kg/day, gentamycin 5 mg/kg/day, and cefotaxime 100 mg/kg/day were given to children with clinical signs of sepsis or proven meningitis. Blood glucose levels were measured using a commercial glucometer. Hypoglycemia was defined as blood glucose below 2.5 mmol/l and treated with an intravenous bolus of 2 ml/kg of 5–10% dextrose followed by feeding or maintenance with 5–10% dextrose. In Guinea, 10% dextrose for intravenous application is not commercially available and had to be reconstituted from 30% dextrose and 5% dextrose. Children with birth asphyxia (BA) were categorized clinically as either mild, moderate, or severe hypoxic-ischemic encephalopathy (HIE) using the Sarnat staging [21]. Seizures were treated with 2mg of rectal diazepam, followed by oral maintenance with 5–10 mg/kg/d of phenobarbital. Radiological investigations, such as chest or abdominal X-rays, echocardiography, or ultrasound were not available on site and therefore rarely obtained due to lacking financial parental resources, restricted opening hours of the external imaging department, and difficulties related to transporting unstable newborns. Full blood counts and levels of C-reactive protein were available 24 hours per day; cerebrospinal fluid cell count with gram stain, blood films, bilirubin, and electrolyte measurements were only available during the daytime and were often not reliable due to equipment malfunction. Blood or liquor cultures were not available.

### Data collection methods

Inclusion criteria were infants admitted to INSE during their newborn period (≤28 days of life) between February 15^th^ and March 16^th^ 2019, who were alive at the time of admission and whose parents had signed the informed consent form. Newborns who died before or at the time of their arrival at INSE and/or whose parents denied processing of their child’s data were excluded from the study. All infants were recruited in this study via convenience sampling. All information related to the hospitalized newborns was documented by physicians and nurses in the standard hospital paper charts. If available, data were collected on maternal history, prenatal and obstetric care, delivery, neonatal conditions, in-hospital management, and laboratory investigations. Provisional diagnoses and presumed causes of death were given by the treating doctor and discussed in the daily morning interdisciplinary staff meeting. Specific definitions used in this study are listed in Table 1. All data recorded for this study were entered in an excel sheet for further analysis.

**Table 1:** Specific definitions of terms used in this study.

Term	Specific definition
**Birth asphyxia (BA)**	Neither Apgar scores nor measurements of umbilical pH were routinely available. Absence of immediate cry after birth and neonatal reanimation (pulmonary or cardiopulmonary resuscitation) were used as criteria to define BA.
**Low birth weight (LBW)**	LBW was defined as by the WHO in the ICD-10 as <2500g (extremely low <1000g, very low 1000-1499g, low 1500-2499g) [22].
**General condition**	Clinical evaluation at admission by the physician assessing the following criteria: skin color, Silverman score, respiratory rate, heart rate, temperature, central and peripheral perfusion, peripheral oxygen saturation, and alertness. Categorization into fair, serious, and critical.
**Gestational age**	Because no gestational age was reported for any newborn, the Ballard Score was used to estimate the gestational age [23].
**Hypoxic-ischemic encephalopathy (HIE)**	Clinical diagnosis using the Sarnat staging: mild (Sarnat grade I), moderate (Sarnat grade II), severe (Sarnat grade III) [21].
**Meningitis**	>30 leucocytes in cerebrospinal fluid, positive gram staining.
**Neurological condition**	Neurological status at admission: Good (normal muscular tone, normal Moro reflex, strong suction, no Sarnat grade, no seizures), fair (reduced or increased muscular tone, weak Moro reflex, weak suction, Sarnat grade I, no seizures), serious (reduced or increased muscular tone, weak/absent Moro reflex, weak/absent suction, Sarnat grade II, seizures present), critical (reduced or increased muscular tone, absent Moro reflex, absent suction, Sarnat grade III, seizures present) [21].
**Prematurity**	Gestational age <37 weeks based on the Ballard Score.
**Presumed neonatal sepsis**	At least one clinical sign of bacterial infection: pallor/cyanosis, dyspnea (nasal flaring, grunting, retractions), tachypnea (respiratory rate >60 breaths/minute), impaired perfusion, temperature instability (<36.5°C or ≥38°C), poor feeding, abdominal distension with or without laboratory findings of infection (elevated C-reactive protein ≥12 mg/l, leukocytosis >30.000/mm^3^ or leukopenia <4.000/mm^3^).
**Respiratory distress**	Using the Silverman Score, respiratory distress was categorized into mild (1–3), moderate (4–6), and severe (>6) [19].

### Data analysis

All analyses were performed using the R system for statistics and graphics version 4.0.4 [24]. Neonatal outcome was categorized into four different, mutually-exclusive states: patients who were cured during hospital stay (cure), deceased during hospital stay (death), were transferred to a different clinic (transfer), or were discharged early for whatever reason (early discharge). For simplicity, the latter two states were combined to a single one (other discharge). We descriptively analyzed how pre-, peri-, and postnatal characteristics may be related to neonatal mortality, stratifying characteristics by neonatal outcome. Frequency and percentage were tabulated for categorical variables, mean and standard deviation for continuous variables. In addition, we fitted a cause-specific Cox proportional-hazards model to assess the associations of patient characteristics at hospital admission with the outcome death (time to in-hospital death), censoring patients with other outcomes (cure or other discharge) at hospital discharge. Explanatory variables in the model were admission weight (in 100 g units), hypothermia (<36.5°C) at admission, neonatal CPR and a dichotomization of general condition and neurological condition into good/ fair vs. serious/critical. Neonatal time to death, cure or other discharge after hospital admission was visualized by cumulative incidence curves. A map of Conakry was produced using the R packages rnaturalearth and rnaturalearthhires, which provided the high-resolution polygon of Guinea, and ggplot2 for plotting the country together with the places of birth of the newborns [25–27].

## Results

**Fig 1:**
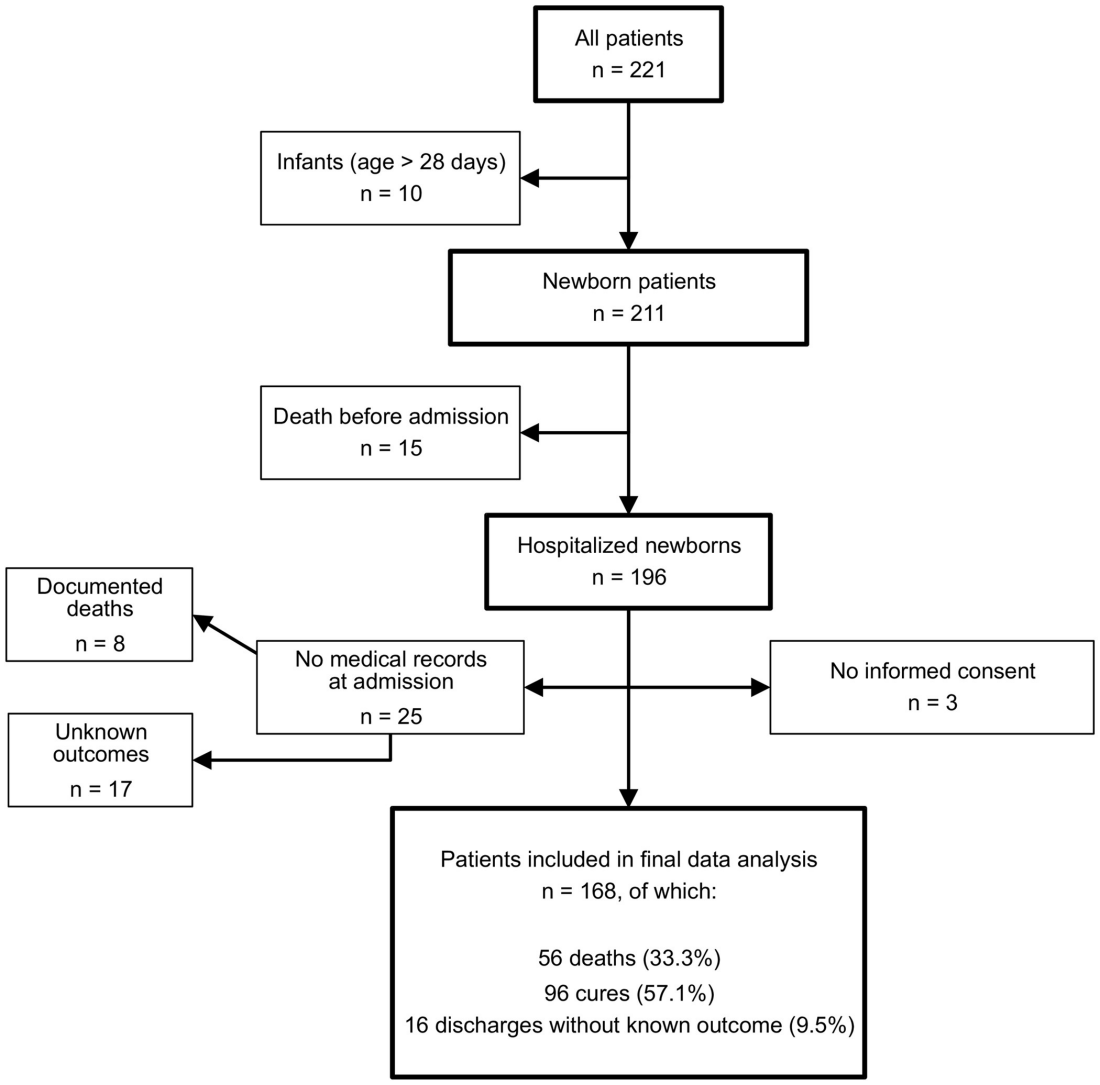
Flow diagram of all included and excluded newborns in the study and their final outcome.

During the study period, 221 infants were brought to the Neonatology Department at INSE (Fig 1). Ten of those were older than 28 days. Of the 211 newborns that were presented at the clinic, 15 were already dead at arrival and eight newborns died during the admission process without further documentation. Additionally, 17 neonates were recorded to be admitted but their outcomes remain unclear due to missing medical forms. Three parents refused to sign the informed consent form. The remaining 168 newborns (79.6%) were included in the study.

### Neonatal mortality

Death occurred in 56 of the included 168 newborns (33.3%, 95%-CI 0.27–0.41). To appreciate the true neonatal mortality rate of patients that were supposed to be treated at INSE, additional rates incorporating the excluded infants were calculated: Considering the eight children that died at admission, the mortality rate rose to 36.4% (64/176, 95%-CI 0.30–0.44). Counting the 15 neonates that died before arrival at INSE, the mortality rate increased to 41.4% (79/191, 95%-CI 0.35–0.48). Out of the included 168 neonates, 96 (57.1%) left the hospital cured; 16 infants (9.5%) were discharged prematurely, either because their parents refused further care (12/168, 7.1%) or because they were transferred to other clinics, e.g., pediatric surgery (4/168, 2.4%). Information about their outcome is not available.

Table 2 depicts the time of death of deceased newborns in relation to their day of life, respectively, the time after admission at INSE. 45 fatalities (80.4%) occurred during the first week of life. The presumed causes of death of the 56 deceased newborns were birth asphyxia (BA) (24/56, 42.9%), prematurity (19/56, 33.9%), sepsis (7/56, 12.5%), congenital malformations (3/56, 5.4%), anaphylactic transfusion reaction (1/56, 1.8%), intestinal perforation (1/56, 1.8%), and anemia (1/56, 1.8%). The three conditions BA, prematurity, and infection accounted for 89.3% of all deaths (50/56). 26.2% (44/168) of newborns required cardiopulmonary resuscitation (CPR) during their hospitalization, 90.9% (40/44) of them deceased. Gender distribution of the fatalities were 64.3% (36/56) males and 35.7% (20/56) females.

**Table 2:** Time of death of deceased newborns in relation to their day of life respectively the time after admission at INSE.

	Time of death	n (%)
**Day of life**	24 hours	13 (23.2%)
	25–72 hours	21 (37.4%)
	Day 4–7	11 (19.6%)
	Day ≥8	11 (19.6%)
**Time after admission**	24 hours	18 (32.1%)
	25–72 hours	23 (41.1%)
	≥73 hours	15 (26.8%)

### Maternal health

Maternal characteristics that were available in at least two-thirds of the cases are shown in Table 3, stratified by neonatal outcome. In general, information regarding maternal health was sparse. Highly relevant aspects for the newborn, such as complications in earlier pregnancies, nutrition, vaccinations, TORCH serology, and medication were missing in almost all cases. Exemplarily, maternal testing for hepatitis B infection was documented in only 25% of cases (3/42, 7.1% hepatitis B positive); testing for maternal diabetes and HIV infection was merely recorded in 41.7% (1/70, 1.4% diabetic) respectively 44.6% of cases (3/75, 4% HIV positive). More than two-thirds (114 women, 67.9%) did not receive the recommended four antenatal care assessments as recommended by the WHO [28]. A large proportion of mothers (43/116, 37.1%) were uneducated.

**Table 3:** The maternal anamnestic characteristics of newborns admitted to INSE for intensive neonatal care, stratified by neonatal outcome (cure or death during hospital stay or other discharge).

		Overall	Cure	Death	Other discharge	Missing n (%)
**n**		168	96	56	16	
**Maternal age in years (mean (SD))**		24.94 (6.46)	25.01 (5.92)	24.96 (7.52)	24.40 (5.75)	11 (6.5)
**Maternal education (%)**	none	43 (37.1)	24 (36.4)	17 (42.5)	2 (20.0)	52 (31.0)
	primary education	19 (16.4)	14 (21.2)	3 (7.5)	2 (20.0)	
	secondary education	38 (32.8)	21 (31.8)	13 (32.5)	4 (40.0)	
	higher education	16 (13.8)	7 (10.6)	7 (17.5)	2 (20.0)	
**Prenatal assessments (%)**	0–1	26 (15.5)	14 (14.6)	10 (17.9)	2 (12.5)	0
	2–3	88 (52.4)	54 (56.2)	27 (48.2)	7 (43.8)	
	4	38 (22.6)	20 (20.8)	13 (23.2)	5 (31.2)	
	>4	16 (9.5)	8 (8.3)	6 (10.7)	2 (12.5)	
**Stillbirth (%)**	0	150 (90.9)	85 (89.5)	51 (94.4)	14 (87.5)	3 (1.8)
	1	11 (6.7)	6 (6.3)	3 (5.6)	2 (12.5)	
	>1	4 (2.4)	4 (4.2)	0 (0.0)	0 (0.0)	
**Gravity (%)**	0–2	90 (53.9)	49 (51.6)	32 (57.1)	9 (56.2)	1 (0.6)
	3–4	45 (26.9)	25 (26.3)	15 (26.8)	5 (31.2)	
	>4	32 (19.2)	21 (22.1)	9 (16.1)	2 (12.5)	
**Parity (%)**	0–2	82 (49.1)	45 (47.4)	28 (50.0)	9 (56.2)	1 (0.6)
	3–4	50 (29.9)	23 (24.2)	21 (37.5)	6 (37.5)	
	>4	35 (21.0)	27 (28.4)	7 (12.5)	1 (6.2)	
**Arterial hypertension (%)**	no	143 (91.7)	78 (88.6)	50 (96.2)	15 (93.8)	12 (7.1)
	yes	13 (8.3)	10 (11.4)	2 (3.8)	1 (6.2)	
**Malaria (%)**	no	51 (42.5)	23 (35.9)	20 (46.5)	8 (61.5)	48 (28.6)
	yes	69 (57.5)	41 (64.1)	23 (53.5)	5 (38.5)	

### Obstetric information

Since all newborns were born outside of INSE, data regarding delivery and adaptation at birth were not available in a substantial proportion of cases. Obstetric information that was available in at least two-thirds of the cases is shown in Table 4, stratified by neonatal outcome. The majority of births were assisted by trained birth attendants in either public or private clinics (149/161, 92.5%; of which 94/161, 58.4% in public and 55/161, 34.2% in private clinics) whereas 7.5% of newborns (12/161) were born at home. Three-quarters of infants were born by uncomplicated vaginal birth (125/166, 75.3%). Poor adaptation, i.e. no immediate cry after birth, needing cardiopulmonary resuscitation (CPR) occurred in 84 infants (50.9%). However, information regarding the duration and measures of CPR was attainable for only 14 children (17.3%). Just one newborn (0.6%) had an Apgar score recorded. Furthermore, notes confirming or negating birth complications were present in 1.7% of cases (3/168). According to their Ballard Score, 46.2% of infants (61/132) were classified as born prematurely. Similarly, 46.2% of admitted newborns (67/145) were born with LBW. Multiple births were the case of 18.5% (31/168) of admitted children; either born as twins (24/168, 14.3%) or triplets (7/168, 4.2%).

**Table 4:** The maternal obstetric and birth characteristics of newborns admitted to INSE, stratified by neonatal outcome (cure or death during hospital stay or other discharge).

		Overall	Cure	Death	Other discharge	Missing n (%)
**n**		168	96	56	16	
**Multiple birth (%)**		31 (18.5)	20 (20.8)	10 (17.9)	1 (6.2)	
**Delivery mode (%)**	normal vaginal birth	125 (75.3)	66 (70.2)	46 (82.1)	13 (81.2)	2 (1.2)
	complicated vaginal birth	15 (9.0)	10 (10.6)	5 (8.9)	0 (0.0)	
	planned caesarean	3 (1.8)	3 (3.2)	0 (0.0)	0 (0.0)	
	emergency caesarean	23 (13.9)	15 (16.0)	5 (8.9)	3 (18.8)	
**Maternal fever (%)**		34 (34.3)	19 (35.8)	15 (44.1)	0 (0.0)	69 (41.1)
**Place of birth (%)**	home birth	12 (7.5)	6 (6.5)	5 (9.4)	1 (6.2)	7 (4.2)
	private clinic	55 (34.2)	33 (35.9)	14 (26.4)	8 (50.0)	
	public clinic	94 (58.4)	53 (57.6)	34 (64.2)	7 (43.8)	
**Sex (%)**	female	61 (36.3)	34 (35.4)	20 (35.7)	7 (43.8)	0
	male	107 (63.7)	62 (64.6)	36 (64.3)	9 (56.2)	
**Birth weight in g (mean (SD))**		2431.79 (908.68)	2522.71 (929.49)	2287.21 (900.45)	2360.00 (778.81)	23 (13.7)
**Birth weight (%)**	extremely low (<1000g)	1 (0.7)	0 (0.0)	1 (2.1)	0 (0.0)	23 (13.7)
	very low (1000-1499g)	27 (18.6)	12 (14.1)	13 (27.7)	2 (15.4)	
	low (1500-2499g)	39 (26.9)	25 (29.4)	10 (21.3)	4 (30.8)	
	normal (> = 2500g)	78 (53.8)	48 (56.5)	23 (48.9)	7 (53.8)	
**Gestational age in weeks (mean (SD))**		35.99 (4.16)	36.38 (3.58)	34.91 (4.88)	38.22 (3.67)	36 (21.4)
**Gestational age (%)**	<32 weeks	23 (17.4)	6 (7.8)	16 (34.8)	1 (11.1)	36 (21.4)
	32–33 weeks	21 (15.9)	14 (18.2)	7 (15.2)	0 (0.0)	
	34–36 weeks	17 (12.9)	14 (18.2)	2 (4.3)	1 (11.1)	
	37–41 weeks	61 (46.2)	38 (49.4)	17 (37.0)	6 (66.7)	
	>41 weeks	10 (7.6)	5 (6.5)	4 (8.7)	1 (11.1)	
**CPR in delivery room (%)**		84 (50.9)	46 (48.4)	31 (55.4)	7 (50.0)	3 (1.8)

### Patient characteristics at admission

**Fig 2:**
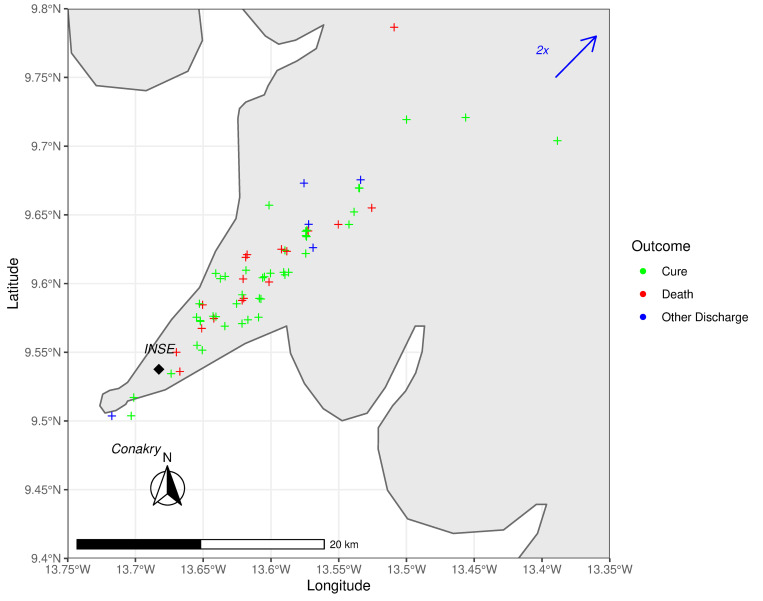
Map of wider Conakry showing the newborns’ points of departure before admission at INSE (black diamond) with their outcome as color-coded crosses (green = cure, red = death, blue = other discharge). Due to inaccuracies in coordinates provided by Google LLC, some crosses appear to be offshore. Two newborns (blue arrow) had a travel distance of 280km.

Detailed patient characteristics at admission that were documented in at least two-thirds of the cases and stratified by neonatal outcomes can be seen in Table 5. All newborns had to be transported prior to admission. 143 infants (87.2%) were referred to INSE by other healthcare facilities, of which only nine (6.3%) provided the families with referral documents. In 21 situations (12.8%) the families searched for medical help autonomously. Most of the children arrived by taxi (94/128, 73.4%), three of them were even transported on motorcycles (2.3%). Just three infants (2.3%) were transported by ambulance. The mean travel distance was 17.6 km (SD 38.5). Travel duration, however, was not reported regularly and depended greatly on the degree of road congestion, having been reported to be as long as three hours. Plotting all points of departure on a map of Conakry, no clear association between increasing distance to INSE and neonatal outcome was found (Fig 2). A substantial number of patients reached INSE in hypothermia (64/167, 38.3%). Patients that would decease later, had lower body temperatures at admission than infants which survived (cured: 37.2 ±1.0°C, deceased: 36.4 ±1.5°C).

**Table 5:** The characteristics at the moment of admission to INSE, stratified by neonatal outcome (cure or death during hospital stay or other discharge).

		Overall	Cure	Death	Other discharge	Missing n (%)
**n**		168	96	56	16	
**Mode of transportation (%)**	ambulance	3 (2.3)	1 (1.4)	2 (4.8)	0 (0.0)	40 (23.8)
	moto-taxi	3 (2.3)	3 (4.2)	0 (0.0)	0 (0.0)	
	private vehicle	31 (24.2)	14 (19.4)	14 (33.3)	3 (21.4)	
	taxi	91 (71.1)	54 (75.0)	26 (61.9)	11 (78.6)	
**Distance to INSE in km (mean (SD))**		17.65 (38.49)	17.90 (40.42)	11.23 (8.61)	37.44 (71.19)	7 (4.2)
**Time of day at admission (%)**	night shift (3 pm—9 am)	118 (70.7)	65 (68.4)	39 (69.6)	14 (87.5)	1 (0.6)
	day shift (9 am—3 pm)	49 (29.3)	30 (31.6)	17 (30.4)	2 (12.5)	
**Age at admission in days (mean (SD))**		3.48 (5.64)	3.25 (5.19)	3.12 (5.46)	6.06 (8.13)	0.0
**Age at admission (%)**	day of life 1	98 (58.3)	54 (56.2)	37 (66.1)	7 (43.8)	0.0
	day of life 2–7	51 (30.4)	32 (33.3)	14 (25.0)	5 (31.2)	
	day of life >7	19 (11.3)	10 (10.4)	5 (8.9)	4 (25.0)	
**Weight at admission in g (mean (SD))**		2380.60 (830.69)	2445.21 (825.07)	2243.93 (862.90)	2477.33 (723.87)	1 (0.6)
**Weight at admission (%)**	<1000g	1 (0.6)	0 (0.0)	1 (1.8)	0 (0.0)	1 (0.6)
	1000-1499g	30 (18.0)	15 (15.6)	14 (25.0)	1 (6.7)	
	1500-2499g	47 (28.1)	27 (28.1)	15 (26.8)	5 (33.3)	
	> = 2500g	89 (53.3)	54 (56.2)	26 (46.4)	9 (60.0)	
**General condition (%)**	fair	64 (38.8)	46 (47.9)	12 (22.2)	6 (40.0)	3 (1.8)
	serious	87 (52.7)	46 (47.9)	32 (59.3)	9 (60.0)	
	critical	14 (8.5)	4 (4.2)	10 (18.5)	0 (0.0)	
**Neurological condition (%)**	good	34 (20.4)	25 (26.0)	4 (7.3)	5 (31.2)	0.6
	fair	43 (25.7)	20 (20.8)	19 (34.5)	4 (25.0)	
	serious	74 (44.3)	45 (46.9)	22 (40.0)	7 (43.8)	
	critical	16 (9.6)	6 (6.2)	10 (18.2)	0 (0.0)	
**Temperature in°C (mean (SD))**		36.97 (1.45)	37.24 (1.40)	36.44 (1.49)	37.20 (1.17)	1 (0.6)
**Temperature in°C (%)**	>38.0	41 (24.6)	27 (28.1)	9 (16.1)	5 (33.3)	1 (0.6)
	<36.5	64 (38.3)	29 (30.2)	31 (55.4)	4 (26.7)	
	36.5–38.0	62 (37.1)	40 (41.7)	16 (28.6)	6 (40.0)	

The majority of newborns were admitted during their first day of life (98/168, 58.3%), 30.4% (51/168) were admitted between postnatal day two and seven, and 11.3% (19/168) were older than one week of postnatal age at admission. Most patients (118/167, 70.7%) were admitted during the afternoon and at night, meaning during the shift (3 pm– 9 am) with reduced medical and nursing staff at INSE. There were no major differences in the proportion of children dying during day shift (34.7%) and night shift (33.1%), respectively. Reasons for admission were respiratory distress (83/168, 49.4%), poor neonatal adaptation (77/168, 45.8%), prematurity (61/132, 46.2%), infection (62/167, 37.1%) among others (54/168, 32.1%; e.g., bleeding 7/168, 4.2% and jaundice 6/168, 3.6%). 101 newborns (61.2%) arrived in serious or critical general condition presenting, for instance, with cyanosis, centralized perfusion, or tachycardia. 90 infants (53.9%) showed symptoms of neurological damage, e.g., absent suction reflex, abnormal muscular tone, or convulsions. Fatal outcome was more frequent in infants arriving in serious/critical general conditions (42/54, 77.8% vs. 48/96, 50%) as well as serious/critical neurological conditions (33/55, 60% vs. 51/96, 53.1%). The mean weight of admitted newborns 2380g (±830g) falls into the category of LBW. Cured newborns had higher weights at admission (2445 ±825g) than deceased children (2243 ±863g). Weight at discharge was documented in only 70.2% (118/168) of the cases, showing that 12.7% (15/118) had lost 0–5% of their weight during hospitalization, 15.2% (18/118) had lost 5–10%, and 14.4% (17/118) more than 10%.

### Patient pathologies and management

A detailed overview of the most common patient pathologies stratified by neonatal outcomes is presented in Table 6. Details regarding prematurity are listed in Table 4. Details of interventions that were documented in at least two-thirds of the cases and stratified by neonatal outcomes can be seen in Table 7.

**Table 6:** The most common patient pathologies of newborns admitted to INSE, stratified by neonatal outcomes (cure or death during hospital stay or other discharge).

		Overall	Cure	Death	Other discharge	Missing n (%)
**n**		168	96	56	16	
**Respiratory distress (%)**		83 (49.4)	41 (42.7)	37 (66.1)	5 (31.2)	0
	Apnea (%)	39 (27.3)	2 (2.3)	36 (83.7)	1 (7.7)	25 (14.9)
**Neurological Disease (%)**		80 (47.6)	43 (44.8)	30 (53.6)	7 (43.8)	0
	Convulsions (%)	17 (10.1)	13 (13.5)	4 (7.1)	0 (0.0)	0
	Birth asphyxia (%)	77 (45.8)	40 (41.7)	30 (53.6)	7 (43.8)	0
**Hypothermia (%)**		64 (38.1)	48 (50.0)	15 (26.8)	1 (6.2)	0
**Infection/sepsis (%)**		62 (37.1)	42 (43.8)	15 (27.3)	5 (31.2)	1 (0.6)
	Early onset sepsis	49 (29.3)	36 (37.5)	10 (18.2)	3 (18.8)	
	Late onset sepsis	11 (6.6)	5 (5.2)	5 (9.1)	1 (6.2)	
	Meningitis	2 (1.2)	1 (1.0)	0 (0.0)	1 (6.2)	
	Probable infection	104 (62.3)	54 (56.2)	39 (70.9)	11 (68.8)	
**Hematological disease (%)**		57 (33.9)	37 (38.5)	17 (30.4)	3 (18.8)	0
	Anemia (%)	19 (11.3)	12 (12.5)	6 (10.7)	1 (6.2)	0
	Jaundice (%)	45 (26.8)	32 (33.3)	11 (19.6)	2 (12.5)	0
**Gastrointestinal disease (%)**		11 (6.5)	4 (4.2)	3 (5.4)	4 (25.0)	0
**Cardiovascular disease (%)**		3 (1.8)	3 (3.1)	0 (0.0)	0 (0.0)	0
**Congenital abnormities (%)**		8 (4.8)	2 (2.1)	5 (8.8)	2 11.8)	0
**Glycemic disturbance (%)**	Hyperglycemia (%)	19 (11.3)	9 (9.4)	8 (14.3)	2 (12.5)	
	Hypoglycemia (%)	6 (3.6)	4 (4.2)	2 (3.6)	0 (0.0)	

**Table 7:** The most common treatments of newborns admitted to INSE, stratified by neonatal outcomes (cure or death during hospital stay or other discharge).

		Overall	Cure	Death	Other discharge	Missing n (%)
**n**		168	96	56	16	
**Vitamin K (%)**		145 (86.3)	83 (86.5)	49 (87.5)	13 (81.2)	0
**Nutrition (%)**	none	5 (4.2)	0 (0.0)	5 (26.3)	0 (0.0)	48 (28.6)
	breastmilk and formula	14 (11.7)	13 (14.1)	0 (0.0)	1 (11.1)	
	breastmilk only	80 (66.7)	63 (68.5)	11 (57.9)	6 (66.7)	
	formula only	21 (17.5)	16 (17.4)	3 (15.8)	2 (22.2)	
**Blood transfusion (%)**		15 (8.9)	7 (7.3)	7 (12.5)	1 (6.2)	0
**Oxygen therapy (%)**		87 (51.8)	37 (38.5)	46 (82.1)	4 (25.0)	0
**Gentamycin (%)**		165 (100.0)	96 (100.0)	54 (100.0)	15 (100.0)	3 (1.8)
**Ampicillin (%)**		161 (99.4)	94 (98.9)	52 (100.0)	15 (100.0)	6 (3.6)
**Caffeine citrate (%)**		36 (21.4)	17 (17.7)	19 (33.9)	0 (0.0)	0
**Cefotaxime (%)**		67 (39.9)	43 (44.8)	18 (32.1)	6 (37.5)	0
**Discontinuity of antibiotic treatment (%)**		114 (67.9)	74 (77.1)	31 (55.4)	9 (56.2)	0
**External heating (%)**		41 (24.4)	15 (15.6)	24 (42.9)	2 (12.5)	0
**Gastric tube (%)**		53 (43.8)	33 (37.5)	17 (73.9)	3 (30.0)	47 (28.0)
**Phototherapy (%)**		25 (16.3)	19 (21.1)	4 (8.3)	2 (13.3)	15 (8.9)
**CPR (%)**		44 (26.2)	3 (3.1)	40 (71.4)	1 (6.2)	0
**Days of hospitalization (mean (SD))**		13.02 (8.19)	16.55 (7.16)	7.53 (6.62)	10.94 (8.06)	2 (1.2)

**Fig 3:**
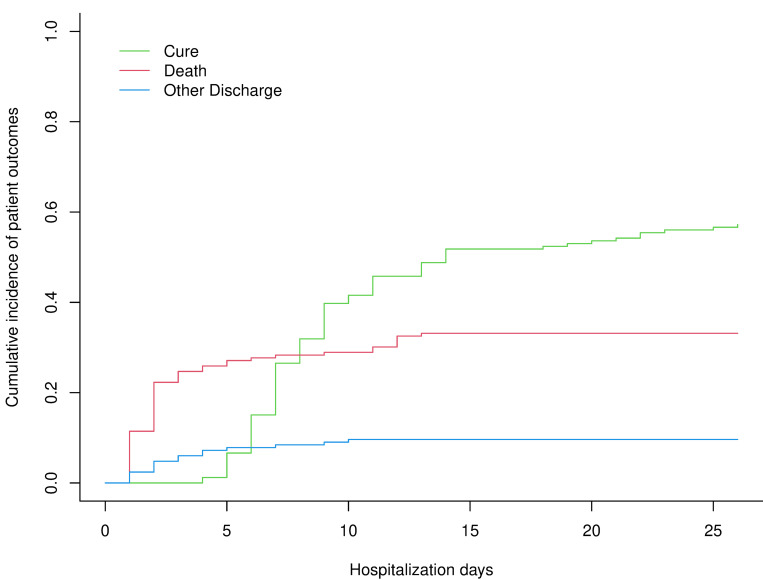
Cumulative incidence plots of neonatal outcome (red = death, green = cure, blue = early discharge).

The mean duration of hospitalization at INSE was 13 days (±8.2 days) with cured newborns staying longer (16.6 ±7.2 days) than those infants who passed away (7.5 ±6.6 days). Most newborns died within the first two days after hospital admission (18 on the first, 19 on the second day), constituting two-thirds of all deaths (37/56, 66.1%) (Fig 3). The majority of newborns (33/37, 89.2%) succumbed to BA (21/37, 56.8%), prematurity (7/37, 18.9%), or infection (5/37, 13.5%) within those two days. The other reasons for death were congenital malformations (2/37, 5.4%), anaphylactic reaction to a blood transfusion (1/37, 2.7%), and anemia (1/37, 2.7%). After day seven of admission, of the nine deaths that were recorded, five were due to prematurity, two due to infection, one due to a congenital malformation, and one due to intestinal occlusion.

All but three newborns (165/168, 98.2%) were multimorbid; 16.1% (27/168) of children suffered from two pathologies, 25.6% (43/168) of the infants were diagnosed with three, 28.6% (48/168) with four, 19.6% (33/168) with five, and 8.3% (14/168) with six or more disease entities. Most commonly, newborns suffered from respiratory distress (83/168, 49.4%). Moderate respiratory distress was found in 52.5% (42/80) of these patients. Severe respiratory distress or impending respiratory failure were found in 23/80 infants (28.8%), whereas mild respiratory distress (10/80, 12.5%) was less common. Of all children with respiratory issues, only 7.2% (6/83) were investigated by chest X-ray. Oxygen was supplied to 51.8% (87/168) of all admitted infants (cured: 42.5%, 37/87; deceased: 52.9%, 46/87) with a mean duration of 1.5 days (±1.3 days; cured: 1.2 ±0.5 days; deceased: 1.8 ±1.8 days). The second most common health issue was prematurity (61/132, 46.2%) with 17.4% of newborns (23/132) with an age below 32 gestational weeks (GW), 15.9% (21/132) between 32 and 33 6/7 GW, and 12.9% (17/132) between 34 and 36 6/7 GW. Of all premature newborns, 29.5% (18/61), remained in the KMC unit with a mean duration of 3.8 days (±1.5 days). BA (77/168, 45.8%) was the third most recorded disease with moderate HIE being most prevalent (51/67, 76.1%; mild HIE 13/67, 19.4%; severe HIE 3/67, 4.4%). More than one-third of newborns suffered from hypothermia at any time during their hospitalization (64/168, 38.1%). However, only 24.4% (41/168) of children were externally heated (cured: 39.0%, 16/41; deceased: 58.5%, 24/41). The fifth most clinically diagnosed disease was neonatal sepsis. 62 newborns (37.1%) showed clinical and laboratory aspects of sepsis with early-onset sepsis (49/167, 29.3%) being more prevalent than late-onset sepsis (11/167, 6.6%). As a measure of precaution, 104 children (62.3%) were categorized as ‘probably septic’ and got antibiotic treatment although they did not fulfill the clinical and laboratory criteria. Only one child (0.6%) was in a well enough condition to be considered non-septic and was not given antibiotics. Hematological diseases were found in 57 newborns (33.9%) with jaundice (45/168, 26.8%) being twice as prevalent as anemia (19/168, 11.3%). 25 children (16.3%) were treated with phototherapy. Congenital abnormalities (8/168, 4.8%) included presumed Down syndrome (*n* = 2), gastroschisis (*n* = 1), encephalocele (*n* = 1), VACTER-syndrome (*n* = 1), cleft palate (*n* = 1), polydactyly (*n* = 1), and club foot (*n* = 1). One newborn had multiple dysmorphic features that could not be linked to a definitive syndrome. 20 infants (11.9%) suffered from microcephaly, an additional seven children (4.2%) had global intrauterine growth retardation, although these data are to be seen in the context of a gestational age only estimated by Ballard score. Six newborns (3.6%) suffered from malnutrition and dehydration. Of all infants that lived long enough to be fed, 41.3% (53/121) were given nutrition by a gastric tube (cured: 62.3%, 33/53; deceased: 32.1%, 17/53). Two-thirds of children exclusively received breastmilk (66.7%, 80/120), 17.5% were given only formula milk (21/120), and 11.7% (14/120) were given both. Four newborns (2.4%) were actively bleeding most likely due to vitamin K deficiency. Vitamin K was administered to 86.3% (145/168) of children at admission (cured: 57.2%, 83/145; deceased: 33.8%, 49/145).

### Indicators for quality of care

As objective indicators for quality of care, six locally available treatment options were evaluated in terms of their exertion: oxygen supply, caffeine citrate, phototherapy, blood transfusions, antibiotics, and external heating. Treatment of respiratory issues: 78.3% (65) of the 83 newborns with respiratory issues were supplied with oxygen. In addition, 22 patients without respiratory distress (25.9%) received oxygen as comfort care. Of the 40 neonates born <34 GW 80% (32/40) were given intravenous caffeine citrate although only 37.5% (15/40) suffered from apneic episodes. On the other hand, none of the 13 infants older than 34 GW with apnea were administered caffeine citrate. In total, 36 newborns were given caffeine citrate, of which 47.2% (17/36) survived and 52.8% (19/36) died. Treatment of jaundice: Out of 45 infants suffering from jaundice, 24 (57.1%) did not receive phototherapy. Only four (8.9%) icteric newborns had their bilirubin levels tested. Treatment of anemia: Nine out of 19 infants with severe anemia (47.4%) were treated with blood transfusions. Peculiarly, six children with normal hemoglobin levels were given blood transfusions. The mean duration between diagnosis of anemia and blood transfusion was 5.5 hours (range 1–23 hours) with 7.3 hours (SD 8.9) for newborns that later deceased, and 4.1 hours (SD 2.9) for infants that survived. Treatment of infection: The vast majority of newborns (98.2%, 165/168) received intravenous antibiotic treatment, either ampicillin plus gentamycin (58.3%, 98/168) or ampicillin plus gentamycin and cefotaxime (39.9%, 67/168). However, only 32.7% of patients (54/165) were given their drugs continuously as planned, the remaining 67.2% (111/165) had single or repetitive gaps in their treatment schedule (cured: 77.1%, 74/96; deceased: 55.4%, 31/56), due to omission of application or unavailability of drugs. Treatment of temperature instability: More than half (38/64, 59.4%) of hypothermic newborns at admission were not externally heated.

### Potential factors associated with neonatal death

We identified two factors present at admission that were significantly associated with neonatal death (Table 8). Newborns arriving in serious/critical general condition had about a fivefold hazard to die than those in good condition (hazard ratio [HR] 5.21, 95%-CI 2.42–11.25, p = <0.0001). Neonates suffering from hypothermia had an approximately twofold hazard than those being admitted with a body temperature of ≥36.5°C (HR 2.00, 95%-CI 1.10–3.65, p = 0.023). A higher admission weight reduced the hazard of neonatal death (HR 0.96 per 100g, 95%-CI 0.92–1.00, p = 0.06), however, this association was just not statistically significant. Interestingly, neither CPR prior to admission nor serious/critical neurological condition seemed to be associated with neonatal mortality.

**Table 8:** Hazard Ratio (HR) estimates from the cause-specific Cox proportional-hazards model on time to death with admission characteristics as explanatory variables.

	HR	95%-CI	z-Value	p-Value
**Admission weight (per 100g)**	0.962	from 0.92 to 1.00	-1.878	0.06
**Hypothermia (<36.5°C) at admission**	2.003	from 1.10 to 3.65	2.268	0.023
**CPR prior to admission**	1.125	from 0.59 to 2.13	-0.361	0.72
**Serious/critical general condition**	5.215	from 2.42 to 11.25	4.21	<0.0001
**Serious/critical neurological condition**	0.601	from 0.33 to 1.09	-1.668	0.095

Patients who left the hospital alive (cured, due to early discharge or transfer to another hospital) were censored at hospital discharge. n = 160 patients were included in the model, of whom 53 died.

Furthermore, three factors occurring during hospitalization were associated with neonatal death (Table 9): Newborns in need of CPR during hospitalization at INSE had a ninefold hazard to decease in comparison to those infants who did not require resuscitation (HR 9.37, 95%-CI 5.01–17.50, p<0.0001). The necessity for oxygen supplementation was also associated with an about twofold hazard of neonatal death (HR 2.57, 95%-CI 1.23–5.38, p = 0.012). The reason for these potentially life-saving interventions being associated with higher hazards of death could be that a newborn in need of such an intervention is already in very serious condition. Contrary to our assumption, discontinuous antibiotic treatment seemed to half the hazard of neonatal death (HR 0.54, 95%-CI 0.31–0.95, p = 0.032).

**Table 9:** Hazard ratio (HR) estimates from the cause-specific Cox proportional-hazards model on time to death with findings during hospitalization as explanatory variables.

	HR	95%-CI	z-Value	p-Value
**Discontinuous antibiotic treatment**	0.540	from 0.31 to 0.95	-2.147	0.032
**CPR during hospitalization**	9.366	from 5.01 to 17.50	7.011	<0.0001
**Oxygen therapy**	2.572	from 1.23 to 5.38	2.508	0.012

Patients who left the hospital alive (cured, due to early discharge or transfer to another hospital) were censored at hospital discharge. *n* = 166 patients were included in the model.

## Discussion

Neonatal mortality remains a significant public health burden in Guinea with 31 deaths per 1,000 live births in 2018. To provide evidence for the implementation of targeted interventions aimed at the reduction of neonatal mortality in accordance with SDG 3.2, we performed a study on neonatal morbidity and mortality, patient characteristics, quality of care, and potential risk factors leading to neonatal death at INSE, the only NICU in Guinea. Our main findings are: The most common disease entities treated at INSE were respiratory distress, prematurity, BA, hypothermia, neonatal sepsis, and jaundice. The most relevant contributors to neonatal death are, consistent with the worldwide pattern, BA, prematurity, and infection [5]. In general, hospitalized newborns at INSE arrived on their day of birth, in serious general and neurological condition, and were shown to be multimorbid. Perinatal and in-hospital quality of care proved to be poor due to insufficient and badly functioning equipment as well as lacking financial and structural resources. Finally, factors significantly associated with in-hospital death included serious/critical general condition and hypothermia at admission, as well as a need for oxygen therapy and in-hospital CPR.

The results represent an important step towards understanding the current challenges in Guinean neonatal care and identifying the most promising and impactful areas for targeted interventions in perinatal medicine. However, the presented results should be interpreted with care considering that this study was conducted in the largest urban agglomeration of Guinea and included only outborn newborns admitted at the only hospital in the country equipped with a NICU facility. Not representative for the whole country, these data, however, are a fundamental mirror of perinatal care and referral practice in the capital area representing 14.7% of the total Guinean population [29].

### Neonatal mortality and causal factors

Of all the newborn infants arriving at INSE with known outcome, 41.4% fatalities were recorded, including 7.9% of patients dead or dying at arrival. The NMR of infants who were alive at admission was 33.3%. Neighboring and economically comparable Guinea-Bissau reported a similarly high NMR of 19.6% at their main NICU [30]. How many newborns have deceased on their way to the hospital and were never presented for confirmation of death remains unknown. Out of all live admitted newborns, one-quarter deceased on their day of birth, which at first seems less than the globally reported proportion of almost one-third [5]. However, if we include the 23 fatalities before and at arrival, this proportion rises to almost half of all newborn deaths. Consistent with previously published data, three-quarters of newborns died within the first week of life [31]. Among the children who died, male newborns were overrepresented compared to female newborns mirroring similar data [32].

The main presumed causes of neonatal death were BA (42.9%), prematurity (33.9%), and infection (12.5%), which is in line with current global data [5, 9, 10]. More than half of admitted newborns (50.9%) have a history of poor postnatal adaptation. These children were presented with moderate to severe encephalopathy at INSE indicating hypoxic brain injury. Contrary to our assumption, compromised neurological functions were not associated with neonatal mortality (Table 8). We argue that this discrepancy results from incomplete postnatal anamnestic information and the difficulty to fully assess BA in low-resource settings lacking techniques to analyze the umbilical blood for its acidosis, the newborn’s blood markers for multiorgan dysfunction and brain imaging as well as cerebral function monitoring [33]. To facilitate diagnosis in developing countries and to prompt neonatal resuscitation interventions without delay, the WHO simply defines BA as “the failure to initiate and sustain breathing at birth” [34]. Initiating basic resuscitation measures providing stimulation, suctioning, and positive pressure ventilation is essential to reduce neonatal mortality in low-income countries [34]. Well performed neonatal CPR requires considerable knowledge, skills, equipment, and continuous training by the birth attendants [35]. Hence, developing feasible strategies to train and equip midwives and physicians for the fast recognition and effective management of BA in this setting is essential [36, 37].

Prematurity as the primary diagnosis contributed to 33.9% of neonatal deaths, which is higher than prior Guinean and global studies reported and can be explained by the selection bias of transferred patients to the INSE, as the only structure caring for LBW and VLBW infants [9, 10, 38, 39]. Premature newborns are highly susceptible to infections, quickly develop severe hypothermia, and often suffer from respiratory distress due to surfactant deficiency and lung immaturity [40, 41] The main reasons for death in premature infants in a similar setting in Ethiopia were found to be respiratory distress syndrome, infections, and BA [42]. We confirmed in this study that the need for oxygen therapy doubled the risk of death as the outcome. In this report, the subgroup of premature newborns was not analyzed for specific morbidities and secondary causes of death. To improve preterm birth outcomes in low-resource settings, the WHO recommends measures such as antenatal corticosteroid therapy for women when preterm labor is imminent, antibiotics for women in preterm labor with preterm prelabor rupture of membranes, routine KMC for newborns weighing less than 2000 g or less, and CPAP therapy for the treatment of respiratory distress syndrome [43]. All these treatment approaches are not yet a standard in Guinea.

Neonatal infection was presumed very likely in 37.1% of cases with either clinical or laboratory aspects of sepsis. Two children had proven meningitis by gram staining of their cerebrospinal fluid. In 12.5% of cases, neonatal infection was the presumed main cause of death. Preventing vertical transmissions of infectious pathogens, ensuring clean birth environments [11], and limiting nosocomial infections [44] is critical in controlling neonatal infections. Implementing microbial laboratories analyzing blood and liquor cultures would greatly aid in the treatment of neonatal infections. The current antibiotic treatment regime at INSE using ampicillin and gentamycin empirically is appropriate according to WHO guidelines [45] and currently available evidence [46], however, its use is overrepresented. The unexpected finding that discontinued antibiotic use reduced the outcome of death by half may be explained by the overuse of antibiotic treatment in 98% of all admissions at the INSE. Therefore, the better outcome in patients with less use of antibiotics reflects probably the fact that these infants did not need antibiotic treatment at all.

Half of the deceased children (51.1%) weighed less than 2500 g. LBW is a major determinant of neonatal mortality [5] and accounts for 60–80% of all neonatal deaths worldwide [47]. LBW is caused by intrauterine growth restriction, premature birth [48], maternal infection, and malnutrition [49]. Identifying and implementing appropriate measures to reduce the causes of LBW should be a priority. Neonatal care units need to be equipped with fully functioning incubators to adequately care for LBW newborns, and KMC units should be increased in capacity and promoted to improve their perceived relevance and reduce early hospital discharge of LBW infants [50, 51].

### Perinatal care and access to postnatal care

This study indicates the dependence of favorable neonatal outcomes on the quality of anti- and perinatal care. Poor general condition of newborns at admission was associated with a five-fold higher hazard of dying compared to those arriving in good condition. All of the admitted newborns at INSE were outborn due to the ongoing reconstruction of the associated maternity ward at Donka University Hospital. Several studies showed that general mortality in neonatal care units caring for outborn and inborn neonates was higher [52, 53] than studies that only included inborn newborns [54, 55]. No study so far analyzed a purely outborn population. Dramatically high with 7.5%, in particular for an urban area, is the rate of children born at home in the absence of any skilled birth attendants. Skilled attendance at birth is inversely related to neonatal mortality [5] and should be a fundamental right for any delivery worldwide. Most of the prenatal history was unknown to the treating physicians at INSE either because mothers did not have the four recommended antenatal care visits (67.9%) or because there was a lack of documentation in the maternal medical history reflecting poor prenatal health services in Guinea. Comprehensive antenatal care has consistently been confirmed to reduce neonatal mortality [56–58]. Improvement in transfer data is an important claim of this study.

One further elusive aspect of this study is the question of how many families refrained from presenting their newborns either because the hospital was too far away from their home/place of birth or they did not have the financial means to cover transportation and hospitalization costs. A descriptive study conducted in Pakistan analyzed the determinants for low utilization of postnatal care and found that, aside from low level of education, lacking awareness, facing transportation issues, and unaffordable cost of healthcare were the most important factors for neglecting postnatal care [59].

Of those admitted at INSE, only 2.3% of children arrived by ambulance, which is half of the previously reported rate from INSE in 2015 [15]. Even in ambulances, respiratory support with oxygen is not available. Therefore, all neonates were transported without placement in incubators for maintaining temperature or surveillance of ongoing treatment by a medical professional. More than one-third of newborns were hypothermic at admission. Without heat loss protection during neonatal transport, a loss of body temperature was shown to be as high as 0.1 to 0.3°C per minute [60]. Our data show a twofold higher hazard of dying among hypothermic newborns at admission. Siqueira and colleagues also showed that hypothermia at admission was significantly associated with early neonatal death even in the presence of high-quality neonatal care [61].

### Quality of care

Although the only NICU in Guinea, the infrastructure and equipment at INSE are insufficient, which diminishes the in-hospital quality of care substantially. Our surrogates of quality of care showed deficiencies in the availability of necessary diagnostic and treatment equipment and usage of existing resources. Lacking essential diagnostic tools coerced healthcare workers into using non-recommended treatment strategies, such as antibiotic treatment for every admitted newborn due to lack of a microbiological laboratory [62, 63]. Almost half of the critically anemic newborns were not given blood transfusions or with a major delay, due to blood collection at the blood bank by the parents themselves.

More than half of hypothermic newborns at admission were not externally heated, and more than one-third of newborns developed hypothermia during their hospitalization. Only one-third of premature newborns were transferred to KMC with their mothers after stabilization in the NICU. These are unfortunate findings since efforts to limit heat loss are important initial steps in the postnatal stabilization of newborns. Extended periods of cold stress have been associated with morbidities, such as respiratory distress [64] and late-onset sepsis [65], as well as death [66], especially in preterm infants and must therefore be prevented rigorously. Often, the only existing bed with overhead heaters at INSE was occupied by other children or inoperable because of power outages. Given these circumstances, local health workers have to adapt and ensure continuous thermal care using alternative strategies, such as skin-to-skin care [67] or placing infants inside plastic bags or aluminum paper [68]. Half of all admitted newborns suffered from respiratory distress underlining the importance of respiratory support and oxygen supply at INSE. Yet, oxygen was not supplied to all children in need, usually, because there were no more available Y-pieces to connect the newborns to the oxygen concentrators. These examples demonstrate that even the theoretically available, but quantitatively underequipped basic resources could not be used to their fullest potential either because the number of children in need exceeded the existing capacity or because their usage was impeded by external factors, such as electric blackouts.

Proof of poor quality of care at INSE and the large chasm between existing and necessary infrastructure to allow for fast and efficient reduction of neonatal mortality illustrate the complexity of challenges that low-income countries are facing in their urge to advance healthcare and reduce mortality. Under resource-deprived circumstances, the financial means typically do not allow for generally recommended strategies to be applied, and locally-customized feasible interventions are required.

### Implications and proposed solutions

These findings show that the current situation for newborns in Guinea is dire and that targeted interventions are urgently needed to achieve the SDG 3.2 of reducing neonatal mortality. Jones et al. proclaim that the majority of child deaths could be prevented by interventions that are feasible for implementation in low-income countries at high levels of population coverage, such as clean delivery, breastfeeding, and antibiotics for sepsis [69]. The high mortality rate of preterm newborns could be diminished by ensuring comprehensive antenatal care, skilled intrapartum assistance, and immediate postnatal care to guarantee appropriate temperature management and nutrition [31]. Birth outside of a neonatal care center with the need to be transferred might have been disadvantageous for newborns in this study. Therefore, conjoining obstetric and neonatal care to allow for emergency delivery where indicated, optimizing immediate postnatal resuscitation maneuvers, and offering timely postnatal interventions, such as antibiotic treatment and maintaining body heat, is crucial [70]. Health services, especially midwifery, must be strengthened to screen for high-risk cases, as identification of fetuses at risk will diminish perinatal complications and reduce neonatal mortality [71]. Furthermore, transfer documents with basic information about maternal health, delivery process and neonatal adaptation are fundamental for the receiving neonatal unit.

It is of utmost importance to facilitate access to INSE by reopening the in-house maternity ward at the Donka University Hospital as soon as possible to bring high-risk deliveries in close to uninterrupted neonatal care at the NICU. The capacities of rooms and beds at the NICU have to be increased, equipment quantitatively and qualitatively to be improved, and a network of medically staffed ambulances equipped with basic devices for neonatal care has to be established.

Easy-to-apply and low-cost interventions like skin-to-skin contact, i.e. KMC, have to be promoted and should be provided to every preterm infant for being highly beneficial and well-tolerated even by critically ill newborns [50]. The recruitment of trained manpower [72], better education [73], supply, support, and supervision [74] for health workers, in particular nurses and midwives, have been shown to be essential in reducing neonatal mortality [75].

This study aims to document the extent of newborn death at the only NICU in Guinea and to galvanize national policy decision-makers, public health experts, and other key stakeholders in the country to realize the pressing need for immediate action in order to reduce neonatal mortality.

### Limitations and future research

Several limitations necessitate cautious interpretation of the results of this study. It was conducted in a single center in the largest urban agglomeration in Guinea. The sample size was small due to the restricted observation period of only one month, which limits the confidence in our findings. All admitted newborns were pre-selected in their severity of disease by being born outside of INSE and only presented in case of relevant symptoms, therefore leading to not negligible selection bias and a non-restricted catchment area. The challenges to accurately diagnose diseases without constantly available and reliable laboratory results were substantial. Treatment options were often not accessible or of inferior quality, and depended largely on the parents’ monetary situation. Additionally, the quality of medical documentation was poor with several untraceable patient histories and insufficiently completed medical forms. Despite these limitations, we believe that our study provides highly relevant information regarding factors for neonatal death in Guinea, especially considering the recentness of data. The results of this study were not aimed at accurately depicting the national epidemiology of neonatal mortality in Guinea. However, as the INSE represents the arrowhead of neonatal medicine in Guinea, this study in the potentially best equipped and staffed neonatal unit unveils a tremendous lack of the Guinean healthcare system regarding perinatal medicine. Future research projects should be designed, but more financial support will be needed to conduct scientifically more robust studies.

## Conclusions

Mortality was shown to be unacceptably high among newborns admitted to the only NICU in Guinea with BA, prematurity, and infection as the main pathways leading to death. Proof of poor quality of in-hospital care accentuates the difficulty to advance healthcare under resource-deprived circumstances. To reduce newborn mortality in this low-resource setting, the priorities should be i) defining strategies to identify fetuses at risk, ii) conjoining obstetric and neonatal care to offer timely postnatal interventions, and iii) improving the resources on all levels, i.e., training of health care professionals, the equipment for diagnostic and treatment aspects as well as the patient documentation system. Additional larger studies are needed to substantiate the findings, facilitate targeted interventions, and give leverage to national health administrators claiming more funding. The situation for neonates in Guinea is nothing short of a human tragedy and if no immediate action is taken more newborns will lose their lives to easily treatable diseases.

## Supporting information

S1 Dataset Dataset as collected from the patient medical records (with maternal age, HIV status, birth weight, and coordinates of birth places removed to protect patients’ identities) and translated from French to English. (PDF)

## Abbreviations

BA: birth asphyxia
CI: confidence interval
CPAP: continuous positive airway pressure
CPR: cardiopulmonary resuscitation
CNERS: National Ethics Committee for Health Research (Comité National d’Ethique pour la Recherche en Santé)
CS: cesarean section
GW: gestational week
HIE: hypoxic-ischemic encephalopathy
HR: hazard ratio
INSE: Institute of Nutrition and Child Health (Institut de Nutrition et de Santé de l’Enfant)
KMC: Kangaroo Mother Care
LBW: low birth weight
MDG: Millennium Development Goals
MSF: Doctors without Borders (Médecins Sans Frontières)
NICU: neonatal intensive care unit
NMR: neonatal mortality rate
SD: standard deviation
SDG: Sustainable Development Goals
UN: United Nations
VLBW: very low birth weight
WHO: World Health Organization
