# Supplementary material for: “Assessing Today for a Better Tomorrow”: An observational cohort study about quality of care, mortality and morbidity among newborn infants admitted to neonatal intensive care in Guinea
Source: PLoS One. 2021 Aug 30;16(8):e0254938. doi: 10.1371/journal.pone.0254938 (PMC8405010; doi:10.1371/journal.pone.0254938)
Supplement: S1 Dataset — (PDF) [file pone.0254938.s001.pdf]

Maternal Age	Maternal Education	CPN Cat.	Stillbirth	Gravity	Parity
30	higher education	2-3	0	3-4	3-4
15	secondary education	2-3	0	0-2	0-2
21	none	2-3	0	3-4	3-4
26	higher education	4	0	0-2	0-2
26	higher education	4	0	0-2	0-2
24		4	0	3-4	0-2
20	none	4	1	3-4	0-2
22	primary education	4	0	0-2	0-2
19		2-3	0	0-2	0-2
18	secondary education	2-3	0	0-2	0-2
30	none	2-3	0	>4	>4
22		4	0	0-2	0-2
25		0-1	0	3-4	>4
22	none	2-3	1	3-4	3-4
22		2-3	1	3-4	3-4
18	primary education	2-3	0	0-2	0-2
18	secondary education	2-3	0	0-2	0-2
35		4	0	3-4	3-4
48	none	2-3	0	>4	>4
35		2-3	1	>4	>4
	none	2-3	0	>4	>4
	none	2-3	0	>4	>4
29		4	0	0-2	0-2
40		0-1	0	>4	>4
19	none	2-3	0	0-2	0-2
25	secondary education	>4	0	0-2	0-2
33		2-3	0	>4	>4
18	none	2-3	0	0-2	0-2
26	none	2-3	0	3-4	3-4
18	secondary education	2-3	0	0-2	0-2
20	none	2-3	0	3-4	3-4
23		0-1	0	0-2	3-4
18	none	2-3	0	0-2	0-2
21		0-1	0	0-2	0-2
28		2-3	0	>4	>4
23	primary education	2-3	>1	3-4	3-4
24	higher education	4	0	0-2	3-4
24	higher education	4	0	0-2	3-4
22	secondary education	0-1	0	0-2	0-2
33	primary education	4	0	>4	>4
30	primary education	2-3	1	>4	>4
19	higher education	4	0	0-2	0-2
16	primary education	2-3	0	0-2	0-2
33	secondary education	2-3	0	>4	>4
33	secondary education	2-3	0	>4	>4
18	secondary education	2-3	0	0-2	0-2
		2-3	0	0-2	0-2
37	higher education	2-3	0	0-2	3-4
37	higher education	2-3	0	0-2	3-4

25		0-1	0	3-4	3-4
27		2-3	0	0-2	0-2
18		2-3	0	0-2	0-2
24	secondary education	2-3	0	0-2	0-2
33	higher education	0-1	0	>4	3-4
		0-1	1	3-4	3-4
28		0-1	0	0-2	0-2
21	primary education	2-3	0	0-2	0-2
20		2-3	0	0-2	0-2
22		>4	0	0-2	0-2
35	secondary education	2-3	1	3-4	3-4
	none	0-1	0	>4	3-4
15	none	2-3	0	0-2	0-2
23	none	2-3	0	3-4	3-4
31	none	>4	0	>4	>4
26	none	2-3	0	>4	>4
17	none	2-3	0	0-2	0-2
17	primary education	4	0	0-2	0-2
28	none	2-3	0	3-4	3-4
40	secondary education	0-1	0	3-4	3-4
22	secondary education	>4	0	3-4	3-4
18	secondary education	0-1	0	0-2	0-2
38	secondary education	>4	0	3-4	0-2
17		2-3	0	0-2	0-2
20		>4	0	3-4	3-4
27	none	2-3	0	3-4	3-4
20	none	4	0	0-2	0-2
25	higher education	2-3	0	0-2	0-2
28	none	4	0	3-4	3-4
22	none	2-3	0	3-4	3-4
33	none	2-3	>1	3-4	3-4
	none	0-1			
32	primary education	2-3	0	>4	>4
32	primary education	0-1	0	>4	>4
30		>4	0	>4	>4
23	secondary education	0-1	0	3-4	3-4
24		2-3	0	3-4	3-4
35	secondary education	4	0	>4	>4
20	secondary education	2-3	0	0-2	0-2
20	secondary education	>4	0	0-2	0-2
19	none	2-3	0	0-2	3-4
18	none	2-3	0	0-2	0-2
20		2-3	0	0-2	3-4
20		2-3	0	0-2	3-4
19		>4	0	0-2	0-2
16		2-3	0	0-2	0-2
16		2-3	0	0-2	0-2
22	none	4		0-2	0-2
19		0-1	0	3-4	3-4
23		4	0	0-2	0-2

30		2-3	0	3-4	3-4
30	none	4	0	3-4	3-4
28	secondary education	2-3	0	0-2	0-2
37		2-3	1	>4	>4
31		2-3	0	>4	3-4
30	higher education	0-1	0	3-4	3-4
	secondary education	4	0	0-2	0-2
28	secondary education	2-3	0	0-2	0-2
25		2-3	0	0-2	0-2
23	none	2-3	0	0-2	0-2
28	none	2-3	1	>4	>4
23	secondary education	2-3	0	3-4	3-4
	higher education	4	0	0-2	3-4
	higher education	4	0	0-2	3-4
38	secondary education	0-1	0	>4	>4
25		4	0	>4	>4
32	none	4	0	3-4	3-4
17	primary education	2-3	0	0-2	0-2
35	none	2-3	0	>4	>4
21	primary education	4	0	0-2	0-2
27	none	2-3	0	0-2	0-2
33	none	2-3	0	3-4	>4
33	none	2-3	0	3-4	>4
33	none	2-3	0	3-4	>4
25	secondary education	>4	0	0-2	0-2
25	secondary education	>4	0	0-2	0-2
28	secondary education	2-3	0	3-4	3-4
19	primary education	0-1		3-4	0-2
17	secondary education	0-1	0	0-2	0-2
17	none	2-3	0	0-2	0-2
20	none	2-3	0	0-2	0-2
32	none	>4	0	>4	>4
20	secondary education	4	0	0-2	0-2
23		4	0	3-4	0-2
		2-3	0	0-2	0-2
29		0-1	0	3-4	3-4
26	none	2-3	0	3-4	3-4
19	secondary education	2-3	0	0-2	0-2
25	secondary education	4	0	0-2	0-2
46		2-3	0	>4	>4
25	primary education	4	0	3-4	3-4
18	primary education	4	0	0-2	0-2
20	primary education	2-3	>1	0-2	>4
20	primary education	2-3	>1	0-2	>4
17	secondary education	>4	0	0-2	0-2
20	none	4	0	0-2	0-2
20	secondary education	2-3	0	0-2	0-2
24		2-3	0	3-4	3-4
25	secondary education	>4	0	0-2	0-2
27	higher education	>4	0	0-2	0-2

18			4	1	0-2	3-4
18			4	1	0-2	3-4
28	primary education		4	0	0-2	0-2
20		>4		0	0-2	0-2
18	secondary education	0-1		0	0-2	0-2
18	secondary education	0-1		0	0-2	0-2
22	none		4	0	3-4	0-2
27			4	0	0-2	0-2
30		2-3		0	3-4	3-4
22		0-1		0	0-2	0-2
30		2-3		0	>4	>4
		2-3		0	3-4	3-4
29	primary education		4	0	>4	>4
31		0-1		0	>4	>4
22		2-3		0	>4	>4
25	secondary education		4	0	0-2	0-2
25	higher education	0-1		0	0-2	0-2
28	higher education	2-3		0	0-2	0-2
19	secondary education	2-3		0	0-2	0-2

Arterial Hypertension	Diabetes	Multiple Birth	Membrane Rupture
no		no	
	no	no	<12 hours
no	no	no	<12 hours
no	no	yes	<12 hours
no	no	yes	<12 hours
no	no	no	12-24 hours
no		no	
no		no	12-24 hours
no		no	12-24 hours
no	no	no	
no		no	12-24 hours
no	no	no	<12 hours
no	no	no	
no		yes	>24 hours
no	no	yes	>24 hours
no	no	no	>24 hours
no	no	no	12-24 hours
yes	no	no	
no		no	<12 hours
no	no	no	<12 hours
no	no	yes	<12 hours
no	no	yes	<12 hours
no	no	no	
no		no	
no		no	
no	no	no	<12 hours
no	no	no	
no		no	>24 hours
no		no	<12 hours
no	no	no	<12 hours
		yes	
		no	
no	no	no	<12 hours
		no	<12 hours
no		no	
yes		no	<12 hours
no	no	yes	
no	no	yes	
no	no	no	12-24 hours
no		no	
no		no	
no	no	no	<12 hours
no		no	12-24 hours
no	no	yes	
no	no	yes	
no	no	no	12-24 hours
no	no	no	<12 hours
no		yes	
no		yes	

no		no	
no		no	
no		no	
no	no	no	
no		no	<12 hours
yes	no	no	<12 hours
		no	<12 hours
no		no	
no		no	
no		no	
no		no	>24 hours
no		no	
no		no	
no	no	no	
yes	yes	no	
no		no	<12 hours
no		no	
no		no	>24 hours
no	no	no	12-24 hours
yes	no	no	
yes	no	no	<12 hours
yes		no	
no	no	no	<12 hours
no	no	no	
no		no	
no	no	no	
no		no	<12 hours
no		no	
no		no	12-24 hours
no		no	12-24 hours
yes		no	
		no	
no		yes	<12 hours
no		yes	<12 hours
no	no	no	
yes		no	
no	no	no	<12 hours
no	no	no	<12 hours
		no	
no	no	no	12-24 hours
no	no	yes	
no	no	no	
no	no	yes	
no	no	yes	
no	no	no	
yes		yes	<12 hours
yes		yes	<12 hours
no	no	no	
no	no	no	12-24 hours
no		no	

no	no	no	
no		no	12-24 hours
no	no	no	12-24 hours
no		no	<12 hours
no		no	
no	no	no	
no		no	
no		no	
no		no	
no		no	
		no	>24 hours
no		no	
no		yes	<12 hours
no		yes	<12 hours
no	no	no	12-24 hours
no	no	no	12-24 hours
no	no	no	12-24 hours
no	no	no	<12 hours
no		no	12-24 hours
no		no	12-24 hours
no		no	
no		yes	
no		yes	
no		yes	
no		yes	<12 hours
no		yes	<12 hours
no		no	<12 hours
no	no	no	<12 hours
no	no	no	12-24 hours
no	no	no	<12 hours
no	no	no	
no	no	no	
no	no	no	
no		no	<12 hours
no		no	
no		no	<12 hours
no		no	
no		no	12-24 hours
no		no	
no	no	no	
no		no	
no		no	
no		yes	
no		yes	
no		no	<12 hours
no		no	
no		no	
no		no	
no	no	no	>24 hours
no	no	no	

no		yes	
no		yes	
no		no	
no	no	no	
		no	
		no	
no	no	no	
no		no	
no		no	
	no	no	<12 hours
no		no	<12 hours
no	no	no	<12 hours
no		no	
yes		no	
no		no	
no		no	
no		no	
yes	no	no	
		no	>24 hours

Delivery Mode	Maternal Birth Fever	Time Of Delivery
	no	night-time (15pm - 8am)
normal vaginal birth		night-time (15pm - 8am)
normal vaginal birth	no	daytime (8am - 15pm)
normal vaginal birth		daytime (8am - 15pm)
normal vaginal birth		
normal vaginal birth	no	night-time (15pm - 8am)
normal vaginal birth	no	night-time (15pm - 8am)
normal vaginal birth	yes	night-time (15pm - 8am)
normal vaginal birth	yes	night-time (15pm - 8am)
normal vaginal birth		night-time (15pm - 8am)
normal vaginal birth	no	night-time (15pm - 8am)
normal vaginal birth		night-time (15pm - 8am)
emergency cesarean		
normal vaginal birth	no	daytime (8am - 15pm)
normal vaginal birth	no	daytime (8am - 15pm)
normal vaginal birth	yes	night-time (15pm - 8am)
normal vaginal birth	no	night-time (15pm - 8am)
planned cesarean		night-time (15pm - 8am)
normal vaginal birth	no	night-time (15pm - 8am)
normal vaginal birth	no	night-time (15pm - 8am)
normal vaginal birth		night-time (15pm - 8am)
normal vaginal birth		night-time (15pm - 8am)
complicated vaginal birth	no	night-time (15pm - 8am)
normal vaginal birth		night-time (15pm - 8am)
normal vaginal birth		night-time (15pm - 8am)
emergency cesarean	no	daytime (8am - 15pm)
emergency cesarean		daytime (8am - 15pm)
normal vaginal birth	no	night-time (15pm - 8am)
normal vaginal birth	yes	night-time (15pm - 8am)
normal vaginal birth	no	night-time (15pm - 8am)
normal vaginal birth		night-time (15pm - 8am)
complicated vaginal birth		night-time (15pm - 8am)
normal vaginal birth	yes	night-time (15pm - 8am)
normal vaginal birth		
emergency cesarean		night-time (15pm - 8am)
normal vaginal birth	yes	daytime (8am - 15pm)
normal vaginal birth	no	night-time (15pm - 8am)
normal vaginal birth	no	night-time (15pm - 8am)
normal vaginal birth	yes	night-time (15pm - 8am)
normal vaginal birth	no	night-time (15pm - 8am)
normal vaginal birth	no	
normal vaginal birth	yes	night-time (15pm - 8am)
normal vaginal birth	yes	daytime (8am - 15pm)
emergency cesarean		daytime (8am - 15pm)
emergency cesarean		daytime (8am - 15pm)
normal vaginal birth		
normal vaginal birth	no	night-time (15pm - 8am)
normal vaginal birth	yes	daytime (8am - 15pm)
normal vaginal birth	yes	daytime (8am - 15pm)

normal vaginal birth		daytime (8am - 15pm)
normal vaginal birth		night-time (15pm - 8am)
emergency cesarean		night-time (15pm - 8am)
complicated vaginal birth	yes	night-time (15pm - 8am)
emergency cesarean	no	night-time (15pm - 8am)
normal vaginal birth		night-time (15pm - 8am)
normal vaginal birth		night-time (15pm - 8am)
normal vaginal birth	yes	night-time (15pm - 8am)
normal vaginal birth		daytime (8am - 15pm)
normal vaginal birth	yes	daytime (8am - 15pm)
emergency cesarean		night-time (15pm - 8am)
normal vaginal birth	yes	night-time (15pm - 8am)
normal vaginal birth	yes	night-time (15pm - 8am)
normal vaginal birth	no	night-time (15pm - 8am)
normal vaginal birth		night-time (15pm - 8am)
normal vaginal birth		night-time (15pm - 8am)
normal vaginal birth	no	night-time (15pm - 8am)
normal vaginal birth	no	daytime (8am - 15pm)
normal vaginal birth	yes	night-time (15pm - 8am)
emergency cesarean	no	
normal vaginal birth	no	night-time (15pm - 8am)
emergency cesarean	no	
normal vaginal birth	no	night-time (15pm - 8am)
normal vaginal birth		night-time (15pm - 8am)
normal vaginal birth		daytime (8am - 15pm)
normal vaginal birth		daytime (8am - 15pm)
normal vaginal birth	no	night-time (15pm - 8am)
emergency cesarean		daytime (8am - 15pm)
normal vaginal birth	yes	night-time (15pm - 8am)
normal vaginal birth	no	night-time (15pm - 8am)
emergency cesarean		daytime (8am - 15pm)
normal vaginal birth		
normal vaginal birth	yes	daytime (8am - 15pm)
normal vaginal birth	yes	daytime (8am - 15pm)
normal vaginal birth		night-time (15pm - 8am)
normal vaginal birth	no	daytime (8am - 15pm)
normal vaginal birth	no	daytime (8am - 15pm)
normal vaginal birth	yes	
normal vaginal birth		night-time (15pm - 8am)
normal vaginal birth	no	night-time (15pm - 8am)
normal vaginal birth	no	
normal vaginal birth	no	
emergency cesarean	yes	
emergency cesarean	yes	
emergency cesarean	no	night-time (15pm - 8am)
normal vaginal birth	yes	
normal vaginal birth	yes	
normal vaginal birth	yes	night-time (15pm - 8am)
normal vaginal birth	yes	
emergency cesarean		night-time (15pm - 8am)

normal vaginal birth		daytime (8am - 15pm)
normal vaginal birth		night-time (15pm - 8am)
normal vaginal birth	no	daytime (8am - 15pm)
normal vaginal birth		night-time (15pm - 8am)
normal vaginal birth	no	
	no	night-time (15pm - 8am)
normal vaginal birth		
emergency cesarean		night-time (15pm - 8am)
complicated vaginal birth		
normal vaginal birth	no	night-time (15pm - 8am)
normal vaginal birth		daytime (8am - 15pm)
emergency cesarean		night-time (15pm - 8am)
planned cesarean	no	
planned cesarean	no	
normal vaginal birth	no	night-time (15pm - 8am)
normal vaginal birth	no	night-time (15pm - 8am)
normal vaginal birth	no	
normal vaginal birth	no	night-time (15pm - 8am)
normal vaginal birth	no	night-time (15pm - 8am)
normal vaginal birth		daytime (8am - 15pm)
normal vaginal birth	no	night-time (15pm - 8am)
normal vaginal birth	no	night-time (15pm - 8am)
normal vaginal birth	no	night-time (15pm - 8am)
normal vaginal birth	no	night-time (15pm - 8am)
complicated vaginal birth		
complicated vaginal birth		
normal vaginal birth		daytime (8am - 15pm)
normal vaginal birth		night-time (15pm - 8am)
normal vaginal birth	no	daytime (8am - 15pm)
emergency cesarean		daytime (8am - 15pm)
normal vaginal birth	no	night-time (15pm - 8am)
normal vaginal birth	no	night-time (15pm - 8am)
normal vaginal birth		night-time (15pm - 8am)
normal vaginal birth	no	night-time (15pm - 8am)
normal vaginal birth		night-time (15pm - 8am)
normal vaginal birth		
normal vaginal birth		night-time (15pm - 8am)
complicated vaginal birth		daytime (8am - 15pm)
emergency cesarean	no	night-time (15pm - 8am)
complicated vaginal birth	yes	night-time (15pm - 8am)
emergency cesarean	yes	night-time (15pm - 8am)
normal vaginal birth		night-time (15pm - 8am)
normal vaginal birth		night-time (15pm - 8am)
normal vaginal birth		night-time (15pm - 8am)
normal vaginal birth	no	night-time (15pm - 8am)
normal vaginal birth	no	night-time (15pm - 8am)
normal vaginal birth	no	daytime (8am - 15pm)
normal vaginal birth	no	
complicated vaginal birth		night-time (15pm - 8am)
normal vaginal birth		night-time (15pm - 8am)

normal vaginal birth	yes	night-time (15pm - 8am)
normal vaginal birth	yes	night-time (15pm - 8am)
normal vaginal birth	no	night-time (15pm - 8am)
normal vaginal birth	yes	daytime (8am - 15pm)
complicated vaginal birth		daytime (8am - 15pm)
complicated vaginal birth		daytime (8am - 15pm)
normal vaginal birth	no	
complicated vaginal birth	yes	night-time (15pm - 8am)
complicated vaginal birth		night-time (15pm - 8am)
normal vaginal birth		night-time (15pm - 8am)
normal vaginal birth		night-time (15pm - 8am)
complicated vaginal birth	no	night-time (15pm - 8am)
normal vaginal birth	no	
emergency cesarean	yes	night-time (15pm - 8am)
normal vaginal birth		night-time (15pm - 8am)
normal vaginal birth	no	daytime (8am - 15pm)
normal vaginal birth		night-time (15pm - 8am)
complicated vaginal birth	no	night-time (15pm - 8am)
normal vaginal birth		daytime (8am - 15pm)

Place Of Birth	Sex	Birth Weight	Gestational Age	Neonatal CPR
private clinic	male	2800	37	yes
private clinic	male	3000	39	yes
private clinic	female	1800	35	no
private clinic	female	1500	32	no
private clinic	female	1200	32	no
private clinic	female	3050		no
private clinic	male	3200		no
private clinic	male	3000	37	yes
public clinic	male	2300		yes
public clinic	male	3100	41	yes
	male	4500	39	yes
public clinic	male	3200	43	yes
	male	3500	39	
public clinic	male	1500	35	yes
public clinic	female	1700	35	yes
public clinic	male	3700		yes
public clinic	female	2900	43	yes
public clinic	female	2900	41	yes
home birth	male			no
private clinic	female	1800		no
public clinic	male	1200	32	no
public clinic	female	1300	32	no
public clinic	male	3100	41	yes
home birth	male	1500	33	no
	male	1300	31	no
public clinic	female	3000	38	yes
private clinic	female	5000	38	no
public clinic	female	1250	28	no
home birth	male		37	no
public clinic	male	2800		no
public clinic	female	800	27	yes
public clinic	female	2600	36	no
private clinic	male	1400	31	no
private clinic	female	1000	29	no
public clinic	male	3000		no
public clinic	female	1500	37	no
public clinic	female			no
public clinic	male			no
public clinic	male	1200	28	no
private clinic	male	3200		no
private clinic	female		38	no
private clinic	male	2940		no
	male	1800	32	no
private clinic	female	2200	33	yes
private clinic	male	2300	33	yes
public clinic	female	3100	43	yes
public clinic	female	2800	37	yes
public clinic	female	1499	28	no
public clinic	female	1200	28	no

public clinic	male	2000	36	yes
home birth	male			no
public clinic	female	1500	33	no
private clinic	male	3000		no
private clinic	female			
public clinic	female	1000	31	no
public clinic	female	1500	31	yes
public clinic	female	1800	35	no
private clinic	female	2000		no
private clinic	female	3200	40	yes
public clinic	female	2200	36	no
private clinic	male		37	yes
public clinic	male	3000	39	yes
private clinic	male	3500		no
private clinic	male	4300	39	yes
public clinic	female	2100	37	yes
public clinic	male	2040		no
public clinic	male	2900		yes
public clinic	male	3650	42	yes
private clinic	female	1450	37	yes
private clinic	male	3420	40	yes
public clinic	female	1300	28	yes
public clinic	male	2700	39	no
public clinic	male	1300	32	no
	female	1500	32	no
private clinic	male	2850		no
private clinic	female	2550	41	yes
public clinic	female	2500	37	yes
public clinic	male	3350	41	yes
private clinic	female	1000		no
public clinic	male	1600	36	no
private clinic	male	3400	39	yes
private clinic	male	2000	37	yes
private clinic	female	3000	37	no
public clinic	male	3000	39	no
public clinic	female	3000	38	yes
public clinic	male	3200	39	no
home birth	male		39	no
public clinic	male	3100	39	no
private clinic	male	3600		yes
home birth	male	1300	31	no
public clinic	male	3100	39	yes
private clinic	male	1700	32	yes
private clinic	female	2000	32	yes
private clinic	female		37	yes
public clinic	male		31	no
public clinic	male		31	no
private clinic	male	3200	39	yes
private clinic	male		32	no
public clinic	female	2600	43	yes

home birth	male	3000	37	no
public clinic	male	3000	43	yes
private clinic	male	3000	41	yes
private clinic	male	1150	35	no
public clinic	male	3000	39	yes
private clinic	male	2500	35	no
private clinic	male	4000	37	yes
public clinic	male	2750	40	yes
public clinic	male	2850	39	yes
private clinic	male	2500	40	no
private clinic	male	2200	35	no
private clinic	female	1800	35	yes
private clinic	male	1200	32	no
private clinic	male	1600	32	no
home birth	male			no
home birth	male		39	no
public clinic	male		40	no
private clinic	male	2030	35	yes
home birth	female		41	yes
public clinic	male	3600	40	yes
public clinic	female	1550	31	yes
public clinic	male	1200	32	no
public clinic	male	1500	32	no
public clinic	male	1300	32	no
public clinic	female	2600	36	no
public clinic	male	2500	36	yes
private clinic	male	3000	40	yes
public clinic	female	3000		yes
public clinic	male	1190	30	yes
public clinic	male	2800	42	yes
public clinic	female	3200		no
private clinic	male	3500	42	yes
public clinic	male		42	yes
public clinic	female			no
	female	1300	28	yes
public clinic	female		29	no
home birth	male			no
private clinic	male	3500		yes
public clinic	female	3500	39	no
private clinic	female	1900	33	yes
private clinic	male	5000	38	yes
public clinic	male	3300	38	yes
public clinic	female	1800	35	yes
public clinic	female	1700	35	yes
public clinic	male	2300	39	yes
public clinic	male	1800		
private clinic	male	2850	39	yes
home birth	female			yes
public clinic	male	2300	39	yes
public clinic	male	2500	39	no

public clinic	male	1150	31	no
public clinic	male	1100	31	no
public clinic	male	3300	41	yes
public clinic	male	3000	40	yes
public clinic	female	1300	31	yes
public clinic	male	1200	31	yes
private clinic	male	3780		no
	female	3560		yes
public clinic	male	3200		no
public clinic	male	2600	43	yes
public clinic	male	2200	37	no
public clinic	male	2700	37	no
public clinic	male	2000	32	no
public clinic	female	1300	29	yes
public clinic	male	4000	38	yes
public clinic	male			yes
public clinic	male	3500	38	yes
public clinic	female	1600		no
public clinic	male	2800		yes

Adm. Time Classification	Transportation	Consultation Reason	General Condition
night-time (3pm - 8am)	private vehicle	Asphyxia	serious
daytime (8am - 3pm)		Asphyxia	serious
		Prematurity	fair
night-time (3pm - 8am)	taxi	Prematurity	fair
night-time (3pm - 8am)	taxi	Prematurity	fair
night-time (3pm - 8am)	taxi	Other	serious
daytime (8am - 3pm)	private vehicle	Other	serious
daytime (8am - 3pm)	taxi	Other	serious
night-time (3pm - 8am)	taxi	Other	serious
night-time (3pm - 8am)	taxi	Asphyxia	serious
night-time (3pm - 8am)	taxi	Respiratory distress	serious
night-time (3pm - 8am)	taxi	Asphyxia	serious
daytime (8am - 3pm)		Other	fair
night-time (3pm - 8am)	taxi	Respiratory distress	fair
night-time (3pm - 8am)	taxi	Respiratory distress	serious
night-time (3pm - 8am)	moto-taxi	Other	critical
night-time (3pm - 8am)	taxi	Asphyxia	critical
night-time (3pm - 8am)	private vehicle	Asphyxia	serious
daytime (8am - 3pm)	taxi	Other	fair
night-time (3pm - 8am)	taxi	Other	fair
night-time (3pm - 8am)	taxi	Prematurity	serious
night-time (3pm - 8am)	taxi	Prematurity	serious
night-time (3pm - 8am)	private vehicle	Asphyxia	serious
night-time (3pm - 8am)	taxi	Prematurity	serious
night-time (3pm - 8am)	private vehicle	Prematurity	
night-time (3pm - 8am)	private vehicle	Asphyxia	critical
night-time (3pm - 8am)	private vehicle	Other	serious
night-time (3pm - 8am)	private vehicle	Prematurity	fair
daytime (8am - 3pm)	taxi	Respiratory distress	serious
night-time (3pm - 8am)	taxi	Other	
night-time (3pm - 8am)	taxi	Prematurity	serious
daytime (8am - 3pm)		Respiratory distress	fair
night-time (3pm - 8am)	taxi	Prematurity	fair
night-time (3pm - 8am)		Prematurity	fair
daytime (8am - 3pm)		Other	serious
daytime (8am - 3pm)	taxi	Prematurity	fair
night-time (3pm - 8am)	private vehicle	Other	serious
night-time (3pm - 8am)	private vehicle	Other	fair
daytime (8am - 3pm)		Prematurity	fair
daytime (8am - 3pm)	taxi	Respiratory distress	serious
daytime (8am - 3pm)	taxi	Other	fair
daytime (8am - 3pm)	taxi	Other	serious
daytime (8am - 3pm)	private vehicle	Prematurity	serious
night-time (3pm - 8am)	taxi	Asphyxia	serious
night-time (3pm - 8am)	taxi	Asphyxia	serious
night-time (3pm - 8am)	taxi	Asphyxia	serious
daytime (8am - 3pm)	taxi	Asphyxia	serious
daytime (8am - 3pm)	taxi	Prematurity	serious
daytime (8am - 3pm)	taxi	Prematurity	serious

night-time (3pm - 8am)	taxi	Asphyxia	critical
night-time (3pm - 8am)	taxi	Other	serious
night-time (3pm - 8am)	taxi	Prematurity	fair
night-time (3pm - 8am)	taxi	Other	fair
night-time (3pm - 8am)	private vehicle	Fever	serious
night-time (3pm - 8am)	taxi	Prematurity	fair
daytime (8am - 3pm)		Prematurity	fair
daytime (8am - 3pm)	taxi	Prematurity	serious
daytime (8am - 3pm)	private vehicle	Other	fair
daytime (8am - 3pm)	taxi	Other	serious
night-time (3pm - 8am)	private vehicle	Prematurity	fair
night-time (3pm - 8am)	private vehicle	Asphyxia	critical
night-time (3pm - 8am)	taxi	Asphyxia	serious
night-time (3pm - 8am)		Other	serious
night-time (3pm - 8am)	taxi	Asphyxia	serious
night-time (3pm - 8am)	taxi	Asphyxia	fair
daytime (8am - 3pm)	taxi	Other	fair
night-time (3pm - 8am)	taxi	Asphyxia	serious
daytime (8am - 3pm)		Asphyxia	
daytime (8am - 3pm)		Asphyxia	fair
night-time (3pm - 8am)		Fever	serious
night-time (3pm - 8am)	moto-taxi	Prematurity	fair
daytime (8am - 3pm)	taxi	Other	serious
daytime (8am - 3pm)	taxi	Prematurity	fair
daytime (8am - 3pm)	taxi	Prematurity	fair
daytime (8am - 3pm)	taxi	Other	critical
night-time (3pm - 8am)	private vehicle	Asphyxia	serious
night-time (3pm - 8am)		Respiratory distress	serious
night-time (3pm - 8am)		Asphyxia	serious
daytime (8am - 3pm)		Other	fair
daytime (8am - 3pm)		Prematurity	fair
night-time (3pm - 8am)		Asphyxia	critical
night-time (3pm - 8am)	taxi	Respiratory distress	serious
night-time (3pm - 8am)	taxi	Respiratory distress	fair
daytime (8am - 3pm)		Other	fair
night-time (3pm - 8am)	taxi	Other	serious
night-time (3pm - 8am)	taxi	Other	serious
night-time (3pm - 8am)	taxi	Fever	serious
night-time (3pm - 8am)	taxi	Other	fair
night-time (3pm - 8am)		Asphyxia	critical
night-time (3pm - 8am)	private vehicle	Prematurity	critical
night-time (3pm - 8am)	taxi	Asphyxia	critical
night-time (3pm - 8am)	taxi	Asphyxia	serious
night-time (3pm - 8am)	taxi	Asphyxia	serious
night-time (3pm - 8am)	private vehicle	Asphyxia	serious
night-time (3pm - 8am)	taxi	Prematurity	fair
night-time (3pm - 8am)	taxi	Prematurity	fair
daytime (8am - 3pm)		Other	fair
daytime (8am - 3pm)	taxi	Other	serious
night-time (3pm - 8am)	taxi	Respiratory distress	serious

night-time (3pm - 8am)	taxi	Other	serious
night-time (3pm - 8am)	taxi	Asphyxia	critical
night-time (3pm - 8am)	taxi	Respiratory distress	serious
night-time (3pm - 8am)	private vehicle	Other	fair
daytime (8am - 3pm)	taxi	Asphyxia	critical
night-time (3pm - 8am)	private vehicle	Respiratory distress	serious
night-time (3pm - 8am)	taxi	Asphyxia	serious
night-time (3pm - 8am)	taxi	Other	serious
daytime (8am - 3pm)		Asphyxia	serious
night-time (3pm - 8am)	private vehicle	Respiratory distress	serious
night-time (3pm - 8am)	taxi	Respiratory distress	serious
night-time (3pm - 8am)	private vehicle	Respiratory distress	serious
daytime (8am - 3pm)		Prematurity	fair
daytime (8am - 3pm)		Prematurity	fair
night-time (3pm - 8am)		Other	fair
night-time (3pm - 8am)		Other	fair
night-time (3pm - 8am)		Other	serious
night-time (3pm - 8am)		Fever	fair
night-time (3pm - 8am)		Other	serious
night-time (3pm - 8am)	taxi	Asphyxia	fair
night-time (3pm - 8am)	taxi	Other	fair
night-time (3pm - 8am)	taxi	Other	fair
night-time (3pm - 8am)	taxi	Other	fair
night-time (3pm - 8am)	taxi	Other	fair
night-time (3pm - 8am)	taxi	Prematurity	fair
night-time (3pm - 8am)	taxi	Fever	serious
night-time (3pm - 8am)	taxi	Asphyxia	serious
daytime (8am - 3pm)		Asphyxia	serious
night-time (3pm - 8am)	taxi	Prematurity	fair
night-time (3pm - 8am)	moto-taxi	Respiratory distress	serious
night-time (3pm - 8am)		Other	fair
night-time (3pm - 8am)	taxi	Asphyxia	serious
night-time (3pm - 8am)	private vehicle	Other	serious
daytime (8am - 3pm)		Other	serious
daytime (8am - 3pm)		Asphyxia	serious
daytime (8am - 3pm)		Prematurity	fair
daytime (8am - 3pm)		Other	serious
daytime (8am - 3pm)	taxi	Other	fair
night-time (3pm - 8am)	taxi	Fever	serious
night-time (3pm - 8am)	private vehicle	Asphyxia	serious
night-time (3pm - 8am)	private vehicle	Other	serious
night-time (3pm - 8am)	private vehicle	Asphyxia	fair
daytime (8am - 3pm)		Prematurity	fair
daytime (8am - 3pm)		Prematurity	fair
night-time (3pm - 8am)	taxi	Other	fair
daytime (8am - 3pm)	taxi	Fever	serious
night-time (3pm - 8am)	taxi	Respiratory distress	serious
night-time (3pm - 8am)	private vehicle	Other	critical
night-time (3pm - 8am)	private vehicle	Asphyxia	serious
night-time (3pm - 8am)	private vehicle	Other	fair

night-time (3pm - 8am)	taxi	Prematurity	fair
night-time (3pm - 8am)	taxi	Prematurity	fair
night-time (3pm - 8am)	taxi	Asphyxia	serious
daytime (8am - 3pm)		Respiratory distress	fair
night-time (3pm - 8am)	ambulance	Asphyxia	serious
night-time (3pm - 8am)	ambulance	Asphyxia	serious
night-time (3pm - 8am)		Fever	serious
night-time (3pm - 8am)	ambulance	Fever	fair
night-time (3pm - 8am)	taxi	Other	fair
night-time (3pm - 8am)	taxi	Respiratory distress	serious
daytime (8am - 3pm)		Other	fair
daytime (8am - 3pm)		Other	fair
night-time (3pm - 8am)	taxi	Respiratory distress	serious
night-time (3pm - 8am)	private vehicle	Asphyxia	serious
night-time (3pm - 8am)	taxi	Asphyxia	serious
night-time (3pm - 8am)	taxi	Other	critical
night-time (3pm - 8am)	private vehicle	Asphyxia	serious
night-time (3pm - 8am)	taxi	Fever	fair
night-time (3pm - 8am)	taxi	Asphyxia	serious

General Condition (binary)	Neurological Condition
serious/critical	serious
serious/critical	fair
good/fair	good
good/fair	fair
good/fair	good
serious/critical	good
serious/critical	fair
serious/critical	serious
serious/critical	serious
serious/critical	serious
serious/critical	serious
serious/critical	serious
good/fair	fair
good/fair	fair
serious/critical	critical
serious/critical	critical
serious/critical	serious
serious/critical	serious
good/fair	fair
good/fair	serious
serious/critical	serious
serious/critical	serious
serious/critical	serious
serious/critical	serious
	serious
serious/critical	critical
serious/critical	good
good/fair	fair
serious/critical	fair
	good
serious/critical	critical
good/fair	fair
good/fair	fair
good/fair	serious
serious/critical	serious
good/fair	good
serious/critical	fair
good/fair	good
good/fair	fair
serious/critical	good
good/fair	good
serious/critical	serious
serious/critical	fair
serious/critical	fair
serious/critical	serious
serious/critical	serious
serious/critical	serious
serious/critical	critical
serious/critical	fair

serious/critical	serious
serious/critical	fair
good/fair	serious
good/fair	serious
serious/critical	serious
good/fair	serious
good/fair	fair
serious/critical	critical
good/fair	good
serious/critical	serious
good/fair	serious
serious/critical	critical
serious/critical	serious
serious/critical	good
serious/critical	serious
good/fair	serious
good/fair	serious
serious/critical	serious
good/fair	good
serious/critical	critical
good/fair	serious
serious/critical	serious
good/fair	fair
good/fair	good
serious/critical	fair
serious/critical	serious
serious/critical	serious
serious/critical	serious
good/fair	good
good/fair	good
serious/critical	fair
serious/critical	serious
good/fair	good
good/fair	good
serious/critical	serious
serious/critical	good
serious/critical	good
good/fair	good
serious/critical	critical
serious/critical	fair
serious/critical	critical
serious/critical	fair
serious/critical	fair
serious/critical	serious
good/fair	fair
good/fair	fair
good/fair	serious
serious/critical	fair
serious/critical	serious

serious/critical	fair
serious/critical	serious
serious/critical	serious
good/fair	serious
serious/critical	critical
serious/critical	critical
serious/critical	critical
serious/critical	serious
serious/critical	fair
serious/critical	fair
serious/critical	serious
serious/critical	critical
good/fair	serious
good/fair	serious
good/fair	good
good/fair	good
serious/critical	serious
good/fair	fair
serious/critical	serious
good/fair	good
good/fair	fair
good/fair	serious
good/fair	fair
good/fair	good
good/fair	good
serious/critical	good
serious/critical	fair
serious/critical	serious
good/fair	fair
serious/critical	serious
good/fair	good
serious/critical	serious
serious/critical	serious
serious/critical	fair
serious/critical	serious
good/fair	good
serious/critical	fair
good/fair	fair
serious/critical	serious
serious/critical	serious
serious/critical	serious
good/fair	serious
good/fair	serious
good/fair	serious
good/fair	fair
serious/critical	good
serious/critical	serious
serious/critical	critical
serious/critical	serious
good/fair	fair

good/fair	fair
good/fair	fair
serious/critical	serious
good/fair	serious
serious/critical	serious
serious/critical	fair
serious/critical	good
good/fair	good
good/fair	serious
serious/critical	serious
good/fair	good
good/fair	fair
serious/critical	serious
serious/critical	serious
serious/critical	serious
serious/critical	good
serious/critical	critical
good/fair	good
serious/critical	serious

Neurological Condition (binary)	Temperature	Age Admission (days)
serious/critical	36.7	1
good/fair	35.5	1
good/fair	36.5	1
good/fair	37	1
good/fair	37.6	1
good/fair	38.5	5
good/fair	38.6	9
serious/critical	38.1	3
serious/critical	36.4	9
serious/critical	35	1
serious/critical	37.5	4
serious/critical	36.6	1
good/fair	36.4	1
good/fair	36.8	1
serious/critical	37	1
serious/critical	37.8	11
serious/critical	37.8	1
serious/critical	36.1	1
good/fair	38.5	4
serious/critical	38.7	2
serious/critical	40.3	1
serious/critical	40.7	1
serious/critical	35.9	1
serious/critical	34.8	1
serious/critical	34	1
serious/critical	34.5	1
good/fair	37.5	1
good/fair	35.8	1
good/fair	37.4	2
good/fair		18
serious/critical	34.9	1
good/fair	35.8	1
good/fair	36.8	1
serious/critical	35	1
serious/critical	38.5	6
good/fair	35.8	2
good/fair	36	25
good/fair	37.7	25
good/fair	36.5	1
good/fair	36.6	22
good/fair	37.2	4
serious/critical	36	7
good/fair	36	1
good/fair	38.2	1
serious/critical	37.9	1
serious/critical	38.7	1
serious/critical	37.8	2
serious/critical	35.5	1
good/fair	35.9	1

serious/critical	35.6	1
good/fair	38.1	5
serious/critical	36.1	1
serious/critical	37.4	3
serious/critical	38.3	9
serious/critical	35.4	1
good/fair	36.7	3
serious/critical	35.4	1
good/fair	37.9	5
serious/critical	38.3	3
serious/critical	37.3	1
serious/critical	39	2
serious/critical	36.2	1
good/fair	39	9
serious/critical	35.5	1
serious/critical	35.3	1
serious/critical	37.3	19
serious/critical	37.7	16
	33.5	1
good/fair	35.6	1
serious/critical	39.1	1
serious/critical	35.4	1
serious/critical	35.5	3
good/fair	36.9	1
good/fair	37	1
good/fair	40.2	16
serious/critical	37	1
serious/critical	37.6	2
serious/critical	36.7	1
good/fair	37.6	22
good/fair	36	1
good/fair	33	1
serious/critical	37.3	2
good/fair	37.1	2
good/fair	38.4	2
serious/critical	35	1
good/fair	40	2
good/fair	38.7	3
good/fair	37.7	2
serious/critical	33.5	1
good/fair	36	1
serious/critical	34.7	1
good/fair	36.2	1
good/fair	34.7	1
serious/critical	35.8	2
good/fair	35.5	2
good/fair	35.3	2
serious/critical	36.1	1
good/fair	37.8	28
serious/critical	36.6	1

good/fair	38.2	1
serious/critical	36.4	1
serious/critical	36.6	1
serious/critical	37.9	1
serious/critical	36.9	1
serious/critical	37	1
serious/critical	38.4	1
serious/critical	37.6	1
good/fair	35.9	1
good/fair	36.6	1
serious/critical	37.1	1
serious/critical	35.6	1
serious/critical	35.8	1
serious/critical	36.6	1
good/fair	38	23
good/fair	38	2
serious/critical	37.7	3
good/fair	36.7	3
serious/critical	37.5	2
good/fair	37.5	1
good/fair	35.6	1
serious/critical	36.8	1
good/fair	37.7	1
good/fair	37	1
good/fair	38.3	2
good/fair	38.9	2
good/fair	37.3	1
serious/critical	35	1
good/fair	36.6	1
serious/critical	38.1	1
good/fair	37.1	15
serious/critical	38.1	1
serious/critical	37.4	2
good/fair	38	6
serious/critical	34	2
good/fair	36.8	2
good/fair	40.1	4
good/fair	37.5	2
serious/critical	40	2
serious/critical	34.8	1
serious/critical	38.1	1
serious/critical	37.4	1
serious/critical	36	1
serious/critical	36.1	1
good/fair	36.4	1
good/fair	38.8	4
serious/critical	39.6	3
serious/critical	38.8	15
serious/critical	35.6	3
good/fair	37.1	2

good/fair	37.3	2
good/fair	37.1	2
serious/critical	35.4	1
serious/critical	39.9	1
serious/critical	36.2	1
good/fair	36.1	1
good/fair	40	4
good/fair	38.4	4
serious/critical	37	2
serious/critical	38.5	1
good/fair	37.2	1
good/fair	37.7	3
serious/critical	36.4	1
serious/critical	36.2	1
serious/critical	36.4	1
good/fair	38.6	28
serious/critical	35	1
good/fair	39.3	18
serious/critical	35.3	1

Admission Weight	Any Infection	Neonatal Infection	Respiratory Distress
2600	suspicion	suspicion	Resp. Distress
3000	suspicion	suspicion	Resp. Distress
1800	suspicion	suspicion	No Resp. Distress
1500	suspicion	suspicion	No Resp. Distress
1450	suspicion	suspicion	No Resp. Distress
2700	yes	Infection	No Resp. Distress
2700	yes	Infection	No Resp. Distress
3000	yes	early onset sepsis	No Resp. Distress
2300	suspicion	suspicion	Resp. Distress
3200	suspicion	suspicion	Resp. Distress
3500	suspicion	suspicion	Resp. Distress
3000	suspicion	suspicion	Resp. Distress
3300	suspicion	suspicion	No Resp. Distress
1500	suspicion	suspicion	No Resp. Distress
1700	suspicion	suspicion	Resp. Distress
3500	suspicion	suspicion	No Resp. Distress
2800	suspicion	suspicion	Resp. Distress
2700	suspicion	suspicion	No Resp. Distress
2000	yes	early onset sepsis	No Resp. Distress
1600	yes	early onset sepsis	No Resp. Distress
1400	yes	early onset sepsis	No Resp. Distress
1500	yes	early onset sepsis	No Resp. Distress
2900	yes	early onset sepsis	Resp. Distress
1500	yes	early onset sepsis	No Resp. Distress
1300	suspicion	suspicion	No Resp. Distress
3000	suspicion	suspicion	Resp. Distress
4100	suspicion	suspicion	Resp. Distress
1200	suspicion	suspicion	No Resp. Distress
2700	yes	early onset sepsis	Resp. Distress
	suspicion	suspicion	No Resp. Distress
800	no	no	No Resp. Distress
2600	suspicion	suspicion	Resp. Distress
1400	suspicion	suspicion	No Resp. Distress
1000	suspicion	suspicion	No Resp. Distress
2000	yes	late onset sepsis	No Resp. Distress
1300	yes	early onset sepsis	No Resp. Distress
1800	yes	late onset sepsis	Resp. Distress
1800	suspicion	suspicion	No Resp. Distress
1000	yes	early onset sepsis	No Resp. Distress
3800	suspicion	suspicion	Resp. Distress
2500	yes	early onset sepsis	No Resp. Distress
2600	suspicion	suspicion	Resp. Distress
1800	suspicion	suspicion	No Resp. Distress
1800	yes	early onset sepsis	No Resp. Distress
2000	suspicion	suspicion	Resp. Distress
2850	yes	early onset sepsis	No Resp. Distress
2300	suspicion	suspicion	Resp. Distress
1200	suspicion	suspicion	Resp. Distress
1100	suspicion	suspicion	Resp. Distress

1900	suspicion	suspicion	Resp. Distress
2700	yes	late onset sepsis	Resp. Distress
1400	suspicion	suspicion	Resp. Distress
2700	suspicion	suspicion	No Resp. Distress
2600	yes	late onset sepsis	Resp. Distress
1400	suspicion	suspicion	No Resp. Distress
1400	yes	early onset sepsis	No Resp. Distress
1800	suspicion	suspicion	Resp. Distress
2100	yes	late onset sepsis	Resp. Distress
3000	yes	early onset sepsis	No Resp. Distress
2100	suspicion	suspicion	No Resp. Distress
2700	yes	early onset sepsis	Resp. Distress
3000	suspicion	suspicion	No Resp. Distress
3200	yes	late onset sepsis	Resp. Distress
4250	suspicion	suspicion	No Resp. Distress
1900	suspicion	suspicion	No Resp. Distress
1600	suspicion	suspicion	No Resp. Distress
2800	suspicion	suspicion	Resp. Distress
3650	suspicion	suspicion	Resp. Distress
1450	suspicion	suspicion	No Resp. Distress
3300	yes	early onset sepsis	Resp. Distress
1300	yes	early onset sepsis	Resp. Distress
2600	yes	early onset sepsis	Resp. Distress
1300	suspicion	suspicion	No Resp. Distress
1500	suspicion	suspicion	No Resp. Distress
2000	yes	late onset sepsis	No Resp. Distress
3300	suspicion	suspicion	Resp. Distress
2300	suspicion	suspicion	Resp. Distress
3400	yes	early onset sepsis	Resp. Distress
1600	suspicion	suspicion	No Resp. Distress
1600	suspicion	suspicion	No Resp. Distress
3400	suspicion	suspicion	No Resp. Distress
2600	yes	early onset sepsis	Resp. Distress
2700	yes	early onset sepsis	Resp. Distress
2700	yes	early onset sepsis	No Resp. Distress
3500	suspicion	suspicion	Resp. Distress
3300	yes	early onset sepsis	No Resp. Distress
3500	yes	early onset sepsis	No Resp. Distress
2600	suspicion	suspicion	No Resp. Distress
2600	suspicion	suspicion	Resp. Distress
1600	suspicion	suspicion	No Resp. Distress
3100	suspicion	suspicion	Resp. Distress
1700	suspicion	suspicion	Resp. Distress
2000	yes	early onset sepsis	No Resp. Distress
3700	suspicion	suspicion	No Resp. Distress
1350	suspicion	suspicion	No Resp. Distress
1100	yes	early onset sepsis	No Resp. Distress
3200	suspicion	suspicion	Resp. Distress
1300	suspicion	suspicion	Resp. Distress
2500	suspicion	suspicion	Resp. Distress

2900	yes	early onset sepsis	No Resp. Distress
3400	suspicion	suspicion	Resp. Distress
2900	suspicion	suspicion	Resp. Distress
1100	suspicion	suspicion	Resp. Distress
3100	yes	early onset sepsis	Resp. Distress
2500	suspicion	suspicion	Resp. Distress
3600	suspicion	suspicion	Resp. Distress
2700	suspicion	suspicion	Resp. Distress
2600	suspicion	suspicion	Resp. Distress
2500	suspicion	suspicion	Resp. Distress
2200	suspicion	suspicion	Resp. Distress
1900	suspicion	suspicion	Resp. Distress
1200	yes	early onset sepsis	No Resp. Distress
1600	suspicion	suspicion	Resp. Distress
2100	yes	late onset sepsis	No Resp. Distress
3100	yes	early onset sepsis	No Resp. Distress
2750	suspicion	suspicion	Resp. Distress
1900	suspicion	suspicion	No Resp. Distress
3200	suspicion	suspicion	Resp. Distress
3650	yes	early onset sepsis	Resp. Distress
1650	suspicion	suspicion	No Resp. Distress
1400	suspicion	suspicion	No Resp. Distress
1520	suspicion	suspicion	No Resp. Distress
1600	suspicion	suspicion	No Resp. Distress
2600	yes	early onset sepsis	No Resp. Distress
2500	yes	early onset sepsis	Resp. Distress
3560	suspicion	suspicion	Resp. Distress
3000			Resp. Distress
1000	suspicion	suspicion	Resp. Distress
2800	yes	early onset sepsis	Resp. Distress
3300	suspicion	suspicion	No Resp. Distress
3500	yes	early onset sepsis	Resp. Distress
3600	suspicion	suspicion	Resp. Distress
2450	yes	early onset sepsis	No Resp. Distress
1200	suspicion	suspicion	No Resp. Distress
1340	suspicion	suspicion	No Resp. Distress
3560	yes	early onset sepsis	No Resp. Distress
3300	suspicion	suspicion	No Resp. Distress
3360	yes	early onset sepsis	No Resp. Distress
1900	suspicion	suspicion	Resp. Distress
3500	yes	early onset sepsis	No Resp. Distress
3300	suspicion	suspicion	Resp. Distress
1800	suspicion	suspicion	No Resp. Distress
1700	suspicion	suspicion	No Resp. Distress
2500	suspicion	suspicion	No Resp. Distress
2200	yes	early onset sepsis	No Resp. Distress
2600	suspicion	suspicion	No Resp. Distress
3150	yes	late onset sepsis	Resp. Distress
2300	suspicion	suspicion	Resp. Distress
2400	yes	early onset sepsis	No Resp. Distress

1120	suspicion	suspicion	No Resp. Distress
1000	suspicion	suspicion	No Resp. Distress
3300	suspicion	suspicion	Resp. Distress
3000	yes	early onset sepsis	Resp. Distress
1100	suspicion	suspicion	Resp. Distress
1200	suspicion	suspicion	Resp. Distress
3900	yes	early onset sepsis	No Resp. Distress
3000	yes	early onset sepsis	No Resp. Distress
3000	yes	early onset sepsis	Resp. Distress
2700	yes	early onset sepsis	Resp. Distress
2700	suspicion	suspicion	No Resp. Distress
2600	yes	early onset sepsis	Resp. Distress
2000	suspicion	suspicion	Resp. Distress
1350	suspicion	suspicion	Resp. Distress
4000	suspicion	suspicion	Resp. Distress
3000	yes	late onset sepsis	Resp. Distress
3500	yes	early onset sepsis	No Resp. Distress
1500	yes	late onset sepsis	No Resp. Distress
2700	suspicion	suspicion	Resp. Distress

Hematological Disease	Anemia	Icterus	Neurological Disease	Convulsions
yes	No Anemia	Icterus	yes	no
no	No Anemia	No Icterus	yes	no
no	No Anemia	No Icterus	no	no
yes	No Anemia	Icterus	no	no
yes	No Anemia	Icterus	no	no
yes	No Anemia	Icterus	no	no
no	No Anemia	No Icterus	yes	no
no	No Anemia	No Icterus	yes	no
yes	Anemia	Icterus	no	no
no	No Anemia	No Icterus	yes	yes
no	No Anemia	No Icterus	yes	yes
no	No Anemia	No Icterus	yes	yes
no	No Anemia	No Icterus	no	no
yes	No Anemia	Icterus	yes	no
no	No Anemia	No Icterus	yes	no
yes	Anemia	No Icterus	no	no
no	No Anemia	No Icterus	yes	no
no	No Anemia	No Icterus	yes	no
no	No Anemia	No Icterus	no	no
no	No Anemia	No Icterus	no	no
yes	No Anemia	Icterus	no	no
yes	No Anemia	Icterus	no	no
no	No Anemia	No Icterus	yes	no
yes	No Anemia	Icterus	no	no
no	No Anemia	No Icterus	no	no
no	No Anemia	No Icterus	yes	no
no	No Anemia	No Icterus	no	no
no	No Anemia	No Icterus	no	no
no	No Anemia	No Icterus	yes	no
no	No Anemia	No Icterus	no	no
no	No Anemia	No Icterus	yes	no
yes	Anemia	Icterus	no	no
no	No Anemia	No Icterus	no	no
yes	No Anemia	Icterus	no	no
yes	No Anemia	Icterus	no	no
no	No Anemia	No Icterus	no	no
no	No Anemia	No Icterus	no	no
no	No Anemia	No Icterus	no	no
no	No Anemia	No Icterus	no	no
no	No Anemia	No Icterus	no	no
yes	Anemia	No Icterus	no	no
yes	No Anemia	Icterus	no	no
yes	Anemia	No Icterus	no	no
no	No Anemia	No Icterus	yes	no
no	No Anemia	No Icterus	yes	no
no	No Anemia	No Icterus	yes	yes
yes	No Anemia	Icterus	yes	no
yes	No Anemia	Icterus	no	no
no	No Anemia	No Icterus	no	no

yes	No Anemia	Icterus	yes	no
yes	No Anemia	Icterus	no	no
yes	No Anemia	Icterus	no	no
yes	No Anemia	Icterus	yes	no
no	No Anemia	No Icterus	no	no
no	No Anemia	No Icterus	no	no
yes	Anemia	Icterus	no	no
no	No Anemia	No Icterus	no	no
no	No Anemia	No Icterus	no	no
no	No Anemia	No Icterus	yes	yes
no	No Anemia	No Icterus	no	no
no	No Anemia	No Icterus	yes	no
no	No Anemia	No Icterus	yes	yes
no	No Anemia	No Icterus	no	no
no	No Anemia	No Icterus	yes	no
no	No Anemia	No Icterus	yes	no
yes	No Anemia	Icterus	no	no
no	No Anemia	No Icterus	yes	yes
no	No Anemia	No Icterus	yes	no
no	No Anemia	No Icterus	yes	no
no	No Anemia	No Icterus	yes	no
yes	Anemia	Icterus	no	no
no	No Anemia	No Icterus	no	no
no	No Anemia	No Icterus	no	no
yes	No Anemia	Icterus	no	no
yes	No Anemia	Icterus	no	no
no	No Anemia	No Icterus	yes	yes
no	No Anemia	No Icterus	yes	no
no	No Anemia	No Icterus	yes	yes
no	No Anemia	No Icterus	no	no
no	No Anemia	No Icterus	no	no
no	No Anemia	No Icterus	yes	yes
no	No Anemia	No Icterus	yes	no
no	No Anemia	No Icterus	no	no
yes	Anemia	No Icterus	no	no
no	No Anemia	No Icterus	yes	no
no	No Anemia	No Icterus	no	no
no	No Anemia	No Icterus	no	no
yes	Anemia	Icterus	no	no
no	No Anemia	No Icterus	yes	no
yes	Anemia	No Icterus	no	no
no	No Anemia	No Icterus	yes	no
yes	Anemia	No Icterus	yes	no
yes	No Anemia	Icterus	yes	no
no	No Anemia	No Icterus	yes	no
yes	No Anemia	Icterus	no	no
yes	No Anemia	Icterus	no	no
no	No Anemia	No Icterus	yes	no
no	No Anemia	No Icterus	no	no
no	No Anemia	No Icterus	yes	no

yes	Anemia	No Icterus	no	no
no	No Anemia	No Icterus	yes	no
no	No Anemia	No Icterus	yes	no
no	No Anemia	No Icterus	no	no
no	No Anemia	No Icterus	yes	no
no	No Anemia	No Icterus	no	no
yes	Anemia	No Icterus	yes	no
no	No Anemia	No Icterus	yes	yes
no	No Anemia	No Icterus	yes	no
yes	Anemia	No Icterus	no	no
yes	No Anemia	Icterus	no	no
no	No Anemia	No Icterus	yes	no
yes	No Anemia	Icterus	no	no
yes	No Anemia	Icterus	no	no
yes	Anemia	Icterus	no	no
yes	Anemia	Icterus	no	no
yes	Anemia	No Icterus	no	no
no	No Anemia	No Icterus	yes	no
no	No Anemia	No Icterus	yes	yes
no	No Anemia	No Icterus	yes	no
yes	No Anemia	Icterus	yes	no
no	No Anemia	No Icterus	no	no
no	No Anemia	No Icterus	no	no
no	No Anemia	No Icterus	no	no
no	No Anemia	No Icterus	no	no
yes	No Anemia	Icterus	yes	no
no	No Anemia	No Icterus	yes	no
no	No Anemia	No Icterus	yes	no
yes	No Anemia	Icterus	yes	no
yes	No Anemia	Icterus	yes	no
no	No Anemia	No Icterus	no	no
no	No Anemia	No Icterus	yes	yes
no	No Anemia	No Icterus	yes	yes
yes	No Anemia	Icterus	no	no
no	No Anemia	No Icterus	yes	no
yes	No Anemia	Icterus	no	no
no	No Anemia	No Icterus	yes	no
no	No Anemia	No Icterus	yes	yes
no	No Anemia	No Icterus	no	no
no	No Anemia	No Icterus	yes	no
no	No Anemia	No Icterus	yes	yes
no	No Anemia	No Icterus	yes	no
yes	No Anemia	Icterus	no	no
no	No Anemia	No Icterus	no	no
no	No Anemia	No Icterus	yes	no
yes	No Anemia	Icterus	no	no
yes	No Anemia	Icterus	yes	yes
no	No Anemia	No Icterus	no	no
no	No Anemia	No Icterus	yes	no
no	No Anemia	No Icterus	no	no

no	No Anemia	No Icterus	no	no
no	No Anemia	No Icterus	no	no
no	No Anemia	No Icterus	yes	no
no	No Anemia	No Icterus	yes	no
yes	Anemia	No Icterus	yes	no
no	No Anemia	No Icterus	yes	no
no	No Anemia	No Icterus	no	no
yes	No Anemia	Icterus	no	no
no	No Anemia	No Icterus	no	no
no	No Anemia	No Icterus	yes	no
no	No Anemia	No Icterus	no	no
yes	No Anemia	Icterus	no	no
no	No Anemia	No Icterus	no	no
no	No Anemia	No Icterus	yes	no
yes	Anemia	No Icterus	yes	no
no	No Anemia	No Icterus	yes	no
no	No Anemia	No Icterus	yes	no
no	No Anemia	No Icterus	no	no
yes	No Anemia	Icterus	yes	no

Asphyxia	Apnea	Hypothermia During Stay	Gastrointestinal	Vitamin K
yes		Hypothermia	no	yes
yes	Apnea	Hypothermia	no	yes
no		Hypothermia	no	yes
no	Apnea	No Hypothermia	no	yes
no		Hypothermia	no	yes
no		No Hypothermia	no	yes
no	No Apnea	No Hypothermia	no	yes
yes	No Apnea	Hypothermia	no	yes
no	No Apnea	Hypothermia	yes	yes
yes		No Hypothermia	no	yes
yes		Hypothermia	no	yes
yes	Apnea	No Hypothermia	no	yes
no	No Apnea	No Hypothermia	no	no
yes	No Apnea	Hypothermia	no	yes
yes	No Apnea	Hypothermia	no	yes
no	No Apnea	No Hypothermia	no	yes
yes	Apnea	No Hypothermia	no	yes
yes		No Hypothermia	no	yes
no	Apnea	No Hypothermia	no	yes
no	No Apnea	No Hypothermia	yes	yes
no	No Apnea	Hypothermia	no	yes
no	No Apnea	Hypothermia	no	yes
yes	No Apnea	Hypothermia	no	yes
no	Apnea	Hypothermia	yes	yes
no	Apnea	Hypothermia	no	yes
yes	Apnea	No Hypothermia	no	no
no	No Apnea	Hypothermia	no	yes
no	No Apnea	Hypothermia	no	yes
yes		No Hypothermia	no	yes
no	No Apnea	No Hypothermia	yes	no
yes	Apnea	Hypothermia	no	no
no		Hypothermia	no	no
no		No Hypothermia	no	yes
no	No Apnea	Hypothermia	no	yes
no	No Apnea	No Hypothermia	no	yes
no	No Apnea	Hypothermia	no	yes
no		No Hypothermia	no	no
no	No Apnea	No Hypothermia	no	yes
no	No Apnea	Hypothermia	no	yes
no	No Apnea	No Hypothermia	no	yes
no	No Apnea	No Hypothermia	no	yes
no	No Apnea	No Hypothermia	yes	yes
no		No Hypothermia	no	yes
yes	No Apnea	Hypothermia	no	yes
yes	No Apnea	Hypothermia	no	yes
yes	No Apnea	Hypothermia	no	yes
yes		No Hypothermia	no	yes
no	No Apnea	Hypothermia	no	yes
no		No Hypothermia	no	yes

yes	No Apnea	No Hypothermia	no	no
no	Apnea	No Hypothermia	no	yes
no	No Apnea	Hypothermia	no	yes
no		No Hypothermia	no	yes
no	No Apnea	No Hypothermia	no	no
no	No Apnea	Hypothermia	no	yes
no	No Apnea	Hypothermia	yes	yes
no	No Apnea	Hypothermia	no	yes
no	No Apnea	No Hypothermia	no	yes
yes	No Apnea	No Hypothermia	no	yes
no		No Hypothermia	no	yes
yes	Apnea	No Hypothermia	no	yes
yes	No Apnea	Hypothermia	no	yes
no	Apnea	No Hypothermia	no	yes
yes	No Apnea	Hypothermia	no	yes
yes	No Apnea	Hypothermia	no	yes
no	No Apnea	No Hypothermia	no	yes
yes	No Apnea	Hypothermia	no	yes
yes	Apnea	No Hypothermia	no	yes
yes	No Apnea	Hypothermia	no	yes
yes	Apnea	No Hypothermia	no	yes
no	Apnea	Hypothermia	no	yes
no		No Hypothermia	no	yes
no	No Apnea	No Hypothermia	no	yes
no	No Apnea	Hypothermia	no	yes
no	Apnea	No Hypothermia	no	yes
yes		No Hypothermia	no	yes
yes	No Apnea	No Hypothermia	no	yes
yes	No Apnea	No Hypothermia	no	yes
no	No Apnea	No Hypothermia	yes	yes
no	No Apnea	No Hypothermia	no	yes
yes	Apnea	Hypothermia	no	yes
yes	No Apnea	No Hypothermia	no	yes
no	No Apnea	No Hypothermia	no	yes
no	No Apnea	No Hypothermia	no	yes
yes	No Apnea	No Hypothermia	no	yes
no	No Apnea	No Hypothermia	no	yes
no	No Apnea	No Hypothermia	no	no
no	No Apnea	No Hypothermia	no	yes
yes	Apnea	No Hypothermia	no	yes
no	Apnea	No Hypothermia	no	yes
yes	Apnea	No Hypothermia	no	no
yes	Apnea	No Hypothermia	no	yes
yes	No Apnea	Hypothermia	no	yes
yes	Apnea	No Hypothermia	no	yes
no	No Apnea	Hypothermia	no	yes
no	No Apnea	No Hypothermia	no	yes
yes		No Hypothermia	no	yes
no	Apnea	No Hypothermia	no	yes
yes	No Apnea	No Hypothermia	no	yes

no		No Hypothermia	yes	yes
yes	No Apnea	No Hypothermia	no	yes
yes	No Apnea	No Hypothermia	no	yes
no	No Apnea	No Hypothermia	no	yes
yes		No Hypothermia	no	yes
no	No Apnea	No Hypothermia	no	yes
yes	No Apnea	No Hypothermia	no	yes
yes	No Apnea	No Hypothermia	no	yes
yes	No Apnea	No Hypothermia	no	yes
no	No Apnea	No Hypothermia	no	yes
no	No Apnea	Hypothermia	no	yes
yes	No Apnea	Hypothermia	no	yes
no	No Apnea	No Hypothermia	no	yes
no	No Apnea	Hypothermia	no	yes
no	No Apnea	Hypothermia	no	no
no	No Apnea	Hypothermia	yes	yes
no	Apnea	No Hypothermia	no	yes
yes		No Hypothermia	no	yes
yes	No Apnea	Hypothermia	no	yes
yes	No Apnea	No Hypothermia	no	yes
yes	Apnea	Hypothermia	no	no
no	No Apnea	Hypothermia	no	yes
no	No Apnea	Hypothermia	no	yes
no	No Apnea	Hypothermia	no	yes
no	No Apnea	Hypothermia	no	yes
yes		No Hypothermia	no	yes
yes	No Apnea	No Hypothermia	no	yes
yes	Apnea	Hypothermia	no	yes
yes	Apnea	Hypothermia	no	yes
yes	No Apnea	Hypothermia	no	yes
no	No Apnea	No Hypothermia	yes	no
yes	No Apnea	No Hypothermia	no	yes
yes	No Apnea	No Hypothermia	no	yes
no	Apnea	No Hypothermia	no	no
yes	Apnea	Hypothermia	no	yes
no	Apnea	Hypothermia	no	yes
no	No Apnea	No Hypothermia	no	no
yes	No Apnea	No Hypothermia	no	yes
no	No Apnea	No Hypothermia	no	yes
yes	Apnea	Hypothermia	no	yes
yes	No Apnea	No Hypothermia	no	yes
yes	No Apnea	Hypothermia	no	yes
no	No Apnea	Hypothermia	no	no
no	No Apnea	Hypothermia	no	no
yes	No Apnea	No Hypothermia	no	yes
no	No Apnea	No Hypothermia	no	no
yes	No Apnea	No Hypothermia	no	yes
no		No Hypothermia	no	no
yes		No Hypothermia	no	yes
no	No Apnea	No Hypothermia	no	yes

no	Apnea	Hypothermia	no	yes
no	Apnea	Hypothermia	yes	yes
yes	No Apnea	No Hypothermia	no	yes
yes	No Apnea	Hypothermia	no	yes
yes	Apnea	Hypothermia	no	yes
yes	Apnea	No Hypothermia	no	yes
no	No Apnea	No Hypothermia	no	no
no	No Apnea	No Hypothermia	no	no
no	No Apnea	No Hypothermia	no	yes
yes	No Apnea	No Hypothermia	no	yes
no	No Apnea	No Hypothermia	no	yes
no	No Apnea	Hypothermia	no	yes
no	Apnea	No Hypothermia	no	yes
yes	Apnea	No Hypothermia	no	yes
yes	Apnea	No Hypothermia	no	yes
yes	No Apnea	No Hypothermia	no	no
yes	Apnea	No Hypothermia	no	yes
no	No Apnea	No Hypothermia	no	no
yes	No Apnea	No Hypothermia	no	yes

Nutrition	Oxygen Therapy	Oxygen Therapy (days)	Gentamycin	Ampicillin
breastmilk	yes	1	yes	yes
	yes	1	yes	yes
breastmilk	no		yes	yes
breastmilk	yes	1	yes	yes
breastmilk	no		yes	yes
breastmilk	no		yes	yes
breastmilk	no		yes	yes
breastmilk	yes	1	yes	yes
breastmilk	yes	1	yes	yes
none	yes	2	yes	yes
breastmilk	yes	1	yes	yes
	no		yes	yes
	yes		yes	yes
breastmilk	no		yes	yes
breastmilk	yes	2	yes	yes
breastmilk	no	1	yes	yes
	yes	2	yes	yes
formula	yes	1	yes	yes
breastmilk	no	1	yes	yes
breastmilk	yes	2	yes	yes
breastmilk	no	1	yes	yes
breastmilk	no	1	yes	yes
formula	yes	1	yes	yes
formula	yes	11	yes	yes
breastmilk	yes		yes	yes
	yes			
formula	yes	1	yes	yes
breastmilk	no	1	yes	yes
breastmilk	yes	2	yes	yes
breastmilk	no			
	yes	3	yes	yes
formula	no		yes	yes
	no		yes	yes
breastmilk & formula	yes	1	yes	yes
breastmilk	no		yes	yes
formula	no		yes	yes
	yes	1	yes	yes
breastmilk	no		yes	yes
breastmilk	no	1	yes	yes
breastmilk	yes	3	yes	yes
breastmilk	no		yes	yes
none	yes	1	yes	yes
	yes	1	yes	yes
breastmilk & formula	no	2	yes	yes
breastmilk	yes	2	yes	yes
breastmilk	no	1	yes	yes
breastmilk	no		yes	yes
breastmilk	yes	3	yes	yes
	yes	1	yes	yes

formula	yes	1	yes	yes
	yes	1	yes	yes
breastmilk	yes	1	yes	yes
breastmilk	no		yes	yes
	yes	1	yes	yes
breastmilk & formula	no	1	yes	yes
formula	no		yes	yes
breastmilk	yes	1	yes	
	no		yes	yes
breastmilk	no	1	yes	no
	no	1	yes	yes
	yes		yes	yes
breastmilk	no		yes	yes
none	yes	8	yes	yes
breastmilk	yes	1	yes	yes
breastmilk	yes	1	yes	yes
breastmilk	no		yes	yes
breastmilk	no		yes	yes
	yes	1	yes	yes
formula	no		yes	yes
	yes	2	yes	yes
formula	yes	1	yes	yes
	yes	1	yes	yes
breastmilk	no		yes	yes
breastmilk & formula	yes	1	yes	yes
breastmilk	yes	1	yes	yes
	yes	2	yes	yes
	yes	2	yes	yes
	yes	2	yes	yes
breastmilk	no		yes	yes
formula	no	1	yes	yes
	yes	1	yes	yes
breastmilk & formula	no	2	yes	yes
breastmilk & formula	no		yes	yes
formula	no		yes	yes
breastmilk	yes	1	yes	yes
breastmilk	no		yes	yes
breastmilk	no		yes	yes
breastmilk	no		yes	yes
	yes	1	yes	yes
	yes	1	yes	yes
	yes	1		
	yes	3	yes	yes
breastmilk	no		yes	yes
formula	no	2	yes	yes
breastmilk	no		yes	yes
breastmilk	no		yes	yes
formula	no		yes	yes
	yes	1	yes	yes
	yes	1	yes	yes

	no		yes	yes
	yes	1	yes	yes
formula	yes	1	yes	yes
breastmilk	no	1	yes	yes
	yes	2	yes	
formula	yes	2	yes	yes
breastmilk	yes	1	yes	yes
breastmilk & formula	yes	1	yes	yes
formula	yes	1	yes	yes
breastmilk	no		yes	yes
breastmilk	yes	1	yes	yes
breastmilk & formula	yes	1	yes	yes
breastmilk	no		yes	yes
breastmilk	no		yes	yes
breastmilk	no		yes	yes
breastmilk	no		yes	yes
	yes	2	yes	yes
	no		yes	yes
breastmilk	no		yes	yes
breastmilk	no	1	yes	yes
breastmilk	no	1	yes	yes
breastmilk	no	1	yes	yes
breastmilk	no		yes	yes
breastmilk	no	1	yes	yes
formula	no		yes	yes
	no		yes	yes
	no	1	yes	yes
	yes	1	yes	yes
breastmilk	yes	1	yes	yes
breastmilk	no		yes	yes
	no		yes	yes
breastmilk	yes	1	yes	yes
breastmilk	yes	1	yes	yes
breastmilk	no		yes	yes
none	yes		yes	yes
breastmilk	no		yes	yes
breastmilk	no		yes	yes
breastmilk & formula	no		yes	yes
breastmilk & formula	no		yes	yes
none	yes	2	yes	yes
breastmilk	yes	1	yes	yes
formula	yes	1	yes	yes
breastmilk & formula	no	1	yes	yes
breastmilk & formula	no	1	yes	yes
	no		yes	yes
breastmilk	no		yes	yes
breastmilk & formula	no		yes	yes
	yes	1	yes	yes
formula	yes	2	yes	yes
formula	no		yes	yes

	yes	1	yes	yes
breastmilk	yes	1	yes	yes
	no	1	yes	yes
breastmilk & formula	yes	1	yes	yes
	yes	1	yes	yes
	yes	1	yes	yes
breastmilk	no		yes	yes
breastmilk	no		yes	yes
breastmilk	yes	1	yes	yes
breastmilk	yes	1	yes	yes
	yes	1	yes	
breastmilk	no	1	yes	yes
	yes	2	yes	yes
	yes	2	yes	yes
	yes	2	yes	yes
breastmilk	yes	1	yes	yes
	yes	2	yes	yes
breastmilk	no		yes	yes
breastmilk	yes	3	yes	yes

Caffeine Citrate	Cefotaxim	Drug Continuation	External Heating	Gastric Tube
no	yes	continuous	no	no
no	no	continuous	no	
no	no	not continuous	no	no
no	no	continuous	yes	yes
yes	no	not continuous	no	yes
no	yes	continuous	no	yes
no	yes	not continuous	no	yes
no	yes	continuous	no	yes
no	yes	continuous	no	no
no	yes	continuous	no	yes
no	yes	continuous	no	no
no	no	continuous	no	
no	no	continuous	no	
no	no	not continuous	no	no
no	no	not continuous	no	no
no	yes	not continuous	no	no
no	no	continuous	no	yes
no	no	not continuous	no	yes
no	no	not continuous	no	yes
no	no	continuous	no	yes
yes	no	not continuous	no	no
yes	no	not continuous	no	no
no	yes	continuous	yes	no
no	yes	not continuous	no	yes
yes	no	not continuous	yes	yes
no	no	continuous	yes	
no	no	continuous	no	no
yes	no	not continuous	yes	yes
no	yes	not continuous	no	yes
no	no	not continuous	no	no
yes	no	not continuous	yes	
no	no	not continuous	no	no
yes	no	not continuous	yes	
yes	no	not continuous	yes	no
no	yes	not continuous	no	yes
yes	no	not continuous	yes	no
no	no	not continuous	no	
no	no	not continuous	no	no
yes	no	not continuous	no	yes
no	yes	not continuous	no	no
no	no	not continuous	no	no
no	yes	continuous	no	
no	no	continuous	no	
yes	no	not continuous	no	yes
yes	no	not continuous	no	yes
no	yes	not continuous	no	yes
no	yes	not continuous	no	yes
yes	no	not continuous	no	yes
yes	no	not continuous	yes	

no	no	not continuous	yes	no
no	yes	not continuous	no	
no	no	not continuous	yes	yes
no	no	not continuous	no	no
no	yes	not continuous	no	
no	no	not continuous	yes	no
no	yes	not continuous	yes	no
no	yes	not continuous	yes	no
no	no	not continuous	no	
no	yes	not continuous	no	no
no	no	continuous	no	yes
no	yes	continuous	no	
no	no	not continuous	no	
no	yes	not continuous	no	yes
no	yes	continuous	yes	no
no	no	continuous	yes	yes
no	no	not continuous	no	yes
no	no	not continuous	no	yes
no	no	not continuous	yes	
no	no	continuous	yes	no
no	yes	not continuous	no	
no	yes	not continuous	no	yes
no	yes	continuous	yes	
yes	no	not continuous	no	no
yes	no	not continuous	no	no
no	yes	not continuous	no	yes
no	yes	continuous	no	no
no	yes	not continuous	no	
no	yes	continuous	no	
no	yes	not continuous	no	no
no	no	not continuous	no	no
no	no	continuous	yes	yes
no	yes	not continuous	no	yes
no	yes	not continuous	no	no
no	no	not continuous	no	yes
no	no	not continuous	no	no
no	no	continuous	no	no
no	no	not continuous	no	no
no	yes	not continuous	no	no
no	no	not continuous	yes	
yes	no	not continuous	yes	
no	no	continuous	yes	
yes	no	continuous	yes	
yes	no	not continuous	no	no
no	no	continuous	no	no
yes	no	continuous	yes	no
yes	no	not continuous	yes	no
no	no	not continuous	no	yes
no	no	not continuous	no	
no	no	not continuous	no	

no	yes	not continuous	no	
no	yes	not continuous	no	
no	yes	not continuous	no	no
no	no	not continuous	no	
no	yes	not continuous	no	
no	yes	continuous	no	yes
no	no	not continuous	no	no
no	yes	not continuous	no	yes
no	yes	continuous	no	no
no	no	not continuous	no	no
no	no	not continuous	no	no
no	no	continuous	yes	yes
yes	no	not continuous	no	no
yes	no	not continuous	no	no
no	yes	continuous	no	yes
no	yes	not continuous	no	no
no	yes	continuous	no	
no	no	continuous	no	yes
no	yes	not continuous	no	yes
no	yes	not continuous	no	yes
yes	no	not continuous	yes	yes
no	yes	not continuous	no	
yes	no	not continuous	no	
yes	no	not continuous	no	
no	no	not continuous	no	no
no	yes	not continuous	no	
no	no	continuous	no	
no	no	continuous	yes	
yes	no	not continuous	yes	yes
no	yes	not continuous	no	no
no	yes	not continuous	no	
no	no	not continuous	no	no
no	yes	not continuous	no	no
no	yes	continuous	no	no
yes	no	not continuous	yes	
yes	no	not continuous	yes	yes
no	yes	not continuous	no	yes
no	yes	not continuous	no	no
no	yes	not continuous	no	no
yes	no	not continuous	yes	
no	yes	not continuous	no	yes
no	no	not continuous	no	no
no	no	continuous	no	yes
no	no	not continuous	no	yes
no	no	not continuous	no	
no	yes	continuous	no	no
no	yes	continuous	no	yes
no	no	not continuous	no	
no	no	not continuous	yes	yes
no	no	not continuous	no	yes

yes	no	continuous	yes	
yes	no	not continuous	yes	yes
no	no	continuous	yes	
no	yes	not continuous	yes	yes
yes	no	continuous	yes	
yes	no	continuous	yes	
no	yes	not continuous	no	no
no	yes	continuous	no	no
no	no	continuous	no	no
no	yes	not continuous	no	no
no	no	not continuous	no	no
no	no	not continuous	no	no
yes	no	continuous	no	no
yes	no	continuous	no	no
no	no	continuous	no	no
no	yes	not continuous	no	no
no	yes	continuous	no	
no	yes	not continuous	no	yes
no	yes	continuous	no	no

Phototherapy	Reanimation	Outcome
yes	no	Cure
	yes	Death
no	no	Cure
	yes	Death
no	no	Cure
yes	no	Other Discharge
no	no	Cure
no	no	Cure
no	no	Cure
no	no	Death
no	no	Cure
	yes	Death
	no	Cure
no	no	Cure
no	no	Cure
no	no	Cure
no	yes	Death
	yes	Cure
	no	Death
no	no	Other Discharge
	no	Cure
yes	no	Cure
no	no	Cure
no	yes	Death
	yes	Death
	yes	Death
no	no	Cure
no	no	Death
no	no	Cure
no	no	Other Discharge
no	yes	Death
no	no	Cure
no	no	Death
no	no	Cure
yes	no	Cure
no	no	Cure
no	no	Death
no	no	Other Discharge
no	no	Cure
no	no	Cure
no	no	Cure
no	yes	Death
	yes	Death
no	no	Cure
no	no	Cure
no	no	Cure
no	no	Cure
no	no	Death
no	yes	Death

no	yes	Cure
no	yes	Death
no	no	Cure
yes	no	Cure
no	no	Other Discharge
no	no	Other Discharge
yes	no	Cure
no	no	Cure
no	no	Cure
no	no	Cure
no	no	Death
no	yes	Death
no	no	Cure
no	yes	Death
no	no	Cure
no	no	Cure
no	no	Cure
	no	Cure
no	yes	Death
no	no	Cure
no	yes	Death
yes	no	Cure
no	no	Death
yes	no	Cure
	no	Cure
yes	yes	Death
no	yes	Death
no	no	Death
no	no	Death
no	no	Other Discharge
	no	Cure
no	yes	Cure
no	no	Cure
no	no	Cure
no	no	Cure
no	no	Cure
no	no	Cure
no	no	Cure
yes	no	Cure
no	yes	Death
no	yes	Death
no	yes	Death
no	no	Death
yes	no	Cure
no	yes	Other Discharge
yes	no	Cure
yes	no	Cure
no	no	Death
no	yes	Death
no	no	Other Discharge

	no	Other Discharge
no	yes	Death
no	no	Other Discharge
no	no	Cure
no	no	Death
no	no	Cure
no	no	Cure
no	no	Cure
no	no	Cure
no	no	Cure
yes	no	Cure
no	no	Cure
yes	no	Cure
yes	no	Cure
yes	no	Cure
no	no	Cure
no	yes	Death
no	no	Other Discharge
no	no	Cure
no	no	Cure
yes	yes	Death
no	no	Cure
no	no	Cure
no	no	Cure
no	no	Cure
yes	no	Death
no	no	Other Discharge
no	yes	Death
no	yes	Death
no	no	Cure
no	no	Cure
no	no	Cure
no	no	Cure
yes	yes	Death
no	yes	Death
no	yes	Death
no	no	Cure
no	no	Cure
no	no	Cure
no	yes	Death
no	no	Cure
no	no	Cure
no	no	Cure
no	no	Cure
no	no	Other Discharge
yes	no	Other Discharge
yes	no	Cure
no	yes	Death
no	no	Death
no	no	Cure

no	yes	Death
no	yes	Death
no	no	Other Discharge
no	no	Cure
no	yes	Death
no	yes	Death
no	no	Cure
yes	no	Cure
no	no	Cure
no	no	Cure
no	no	Death
yes	no	Cure
no	yes	Death
no	yes	Death
no	yes	Death
no	no	Cure
	yes	Death
no	no	Cure
yes	no	Cure
